# Modernized Machine Learning Approach to Illuminate Enzyme Immobilization for Biocatalysis

**DOI:** 10.1021/acscentsci.3c00757

**Published:** 2023-09-27

**Authors:** Hong Wei, Joseph P. Smith

**Affiliations:** Process Research & Development, MRL, Merck & Co., Inc., West Point, Pennsylvania 19486, United States

## Abstract

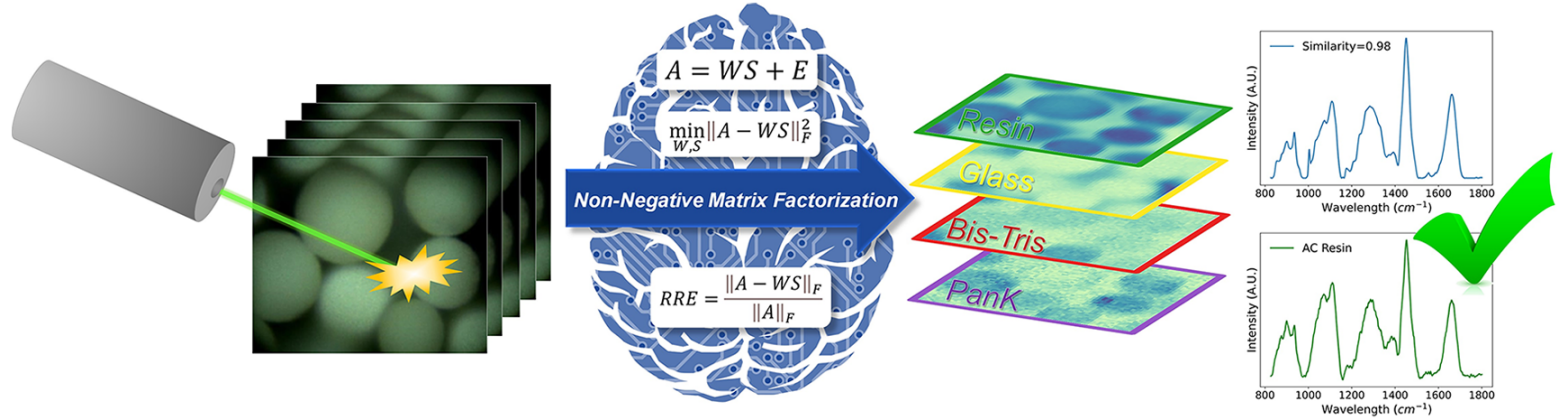

Biocatalysis is an established technology with significant application in the pharmaceutical industry. Immobilization of enzymes offers significant benefits for commercial and practical purposes to enhance the stability and recyclability of biocatalysts. Determination of the spatial and chemical distributions of immobilized enzymes on solid support materials is essential for an optimal catalytic performance. However, current analytical methodologies often fall short of rapidly identifying and characterizing immobilized enzyme systems. Herein, we present a new analytical methodology that combines non-negative matrix factorization (NMF)—an unsupervised machine learning tool—with Raman hyperspectral imaging to simultaneously resolve the spatial and spectral characteristics of all individual species involved in enzyme immobilization. Our novel approach facilitates the determination of the optimal NMF model using new data-driven, quantitative selection criteria that fully resolve all chemical species present, offering a robust methodology for analyzing immobilized enzymes. Specifically, we demonstrate the ability of NMF with Raman hyperspectral imaging to resolve the spatial and spectral profiles of an engineered pantothenate kinase immobilized on two different commercial microporous resins. Our results demonstrate that this approach can accurately identify and spatially resolve all species within this enzyme immobilization process. To the best of our knowledge, this is the *first* report of NMF within hyperspectral imaging for enzyme immobilization analysis, and as such, our methodology can now provide a new powerful tool to streamline biocatalytic process development within the pharmaceutical industry.

## Abstract 2

Novel machine learning approach for spatial resolution and molecular identification of immobilized enzymes for biocatalysis. Our new NMF-based technology offers an enhanced machine learning analysis.

## 1 Introduction

Biocatalysis has led to tremendous innovation in modern organic chemistry by facilitating the synthesis of complex molecules via sustainable and efficient processes, both in academic research and the chemical and pharmaceutical industries.^1−4^ The past two decades have seen the emergence of directed evolution as a powerful molecular biology tool for tailoring enzymes to operate under mild reaction conditions with optimized catalytic activities.^5−7^ In addition, directed enzyme evolution can be leveraged to catalyze new chemical reactions that do not occur in nature.^8,9^ The 2018 Nobel Prize in Chemistry in part acknowledged Dr. Frances H. Arnold for her pioneering contributions to the directed evolution of enzymes.^10^ Recent advancements in directed enzyme evolution have not only enabled the use of individual enzymes as catalysts in single-reagent systems but have also significantly contributed to the development of highly efficient multistep enzymatic cascades in one-pot reactions.^11^ These advances can further help transform pharmaceutical manufacturing, as evidence by, for instance, the fully biocatalyst-driven synthesis of the HIV treatment islatravir.^12^

Industrial implementation of biocatalysis via evolved enzymes can often be hindered by limited durability under operational and storage conditions, high production cost, and lack of reusability.^13^ Immobilization of the respective enzyme is a promising approach to overcome these limitations.^14^ Biocatalyst immobilization methods can be classified into three categories: entrapment by confining an enzyme within a network or matrix, adsorption or covalent binding to a solid carrier, and intermolecular bond formation between enzymes by cross-linking.^15^ Among all methods, carrier-bound immobilization achieved by covalent linkage of enzymes to a solid support is highly desirable since it can minimize enzyme contamination in the final product and facilitate straightforward product separation.^16^ Furthermore, the immobilization of enzymes can also offer additional benefits that include easy handling, repeated use in reaction cycles, and improved recovery from the reaction mixture.

Direct visualization of the enzyme distribution within solid-supported materials is crucial for the development and optimization of enzyme immobilization processes and evaluating its reaction performance.^17^ The selection of an ideal solid carrier material is essential to ensuring a homogeneous distribution of immobilized enzymes. Moreover, accessing the potential for enzyme detachment is important, as it can impact the distribution of enzyme within the support material. A robust analytical technology capable of measuring the distribution of immobilized enzymes on porous supports is highly desired. As the integration of two or more types of enzymes gains prevalence to facilitate synthetic cascades, the accurate localization and spatial distribution of individual enzymes within the porous support become increasingly imperative for optimal performance.

A robust methodology that can provide both spatial and chemical information is essential for facilitating the implementation of biocatalysis in practical applications. Fluorescence microscopy is the most widely used analytical method to track the trajectory of enzymes within the support materials. However, the selection of fluorescent labels and the maintenance of signal stability remains challenging.^18^ Raman microscopy can provide label-free vibrational fingerprint information on both the enzyme and support materials.^19^ Moreover, Raman hyperspectral imaging offers the capability to simultaneously image multiple chemical species, with spatial information collected in the *X–Y* plane, while the spectral information is represented in the *Z* plane.^20−24^ However, the complex background and potential peak overlapping of Raman hyperspectral imaging data limit its widespread application.

Machine learning has exhibited remarkable performance in analyzing vast spectroscopic data from biological samples with complex backgrounds.^25−29^ Specifically, non-negative matrix factorization (NMF) is an unsupervised machine learning technique that is frequently employed for unmixing pure, individual chemical species present within mixtures.^30−32^ NMF operates by factorizing an original non-negative data matrix into both non-negative basis vectors and corresponding weights via multiplicative algorithms that minimize the norm of the difference matrix between the original data matrix and its approximate reconstruction.^33−35^ NMF is advantageous over other decomposition methods because the resolved components reflect true spectra and provide direct chemical information about a mixture rather than variance-based indirect loadings. However, selecting the appropriate number of components to construct an NMF model while avoiding over- or underfitting remains a challenge.

Herein, a new analytical methodology was developed and applied using NMF in conjugation with Raman hyperspectral imaging for the analysis of immobilized biocatalysts. The immobilized enzyme studied in this work was evolved pantothenate kinase (PanK), which was directly utilized for the manufacturing of the HIV treatment islatravir.^12^ Specifically, Raman hyperspectral imaging data was collected—using both 532 and 785 nm excitation—on PanK immobilized to acrylamide (AC) and methacrylate (ME) based resin beads. Previous work demonstrated that principal component analysis (PCA) can provide concentration profiles but not sufficiently resolved spectra for direct comparison with reference data.^36^ Multivariate curve resolution-alternating least-squares (MCR-ALS) can provide concentration profiles and relevant spectra to unmix species involved in enzyme immobilization;^27^ however, optimal MCR-ALS model selection and subsequent species identification remains challenging.

In this work, the determination of the optimal number of components needed for NMF model construction was successfully accomplished. This new approach to determining the optimal NMF model was accomplished by first constructing independent NMF models containing one-ten components and subsequently comparing the NMF-resolved spectra with that of reference materials. Quantitation of NMF-resolved spectra and reference spectra was achieved by utilizing a cosine similarity score as the evaluation matrix,^32^ which allows for determination of the optimal NMF model from the ten independent models with varying numbers of components. We analyzed an exhaustive subset of samples, including unaltered immobilized enzymes and cross-sectional representations of immobilized enzyme prepared by microtome sectioning of the solid supports. In total, we herein report the results from NMF applied to five different Raman hyperspectral imaging data sets collected across PanK immobilized in diverse resins. To the best of our knowledge, this is the *first* report of NMF with hyperspectral imaging for enzyme immobilization analysis. This report can therefore allow a key technology that uses machine learning and hyperspectral imaging to be introduced for enhancing biocatalysis and direct enzyme evolution efforts across the scientific community at large.

## 2 Materials and Methods

### 2.1 Sample Preparation

The N-terminal hexahistidine sequence of the evolved pantothenate kinase (PanK) was attached to a Ni-nitrilotriacetic acid immobilized metal affinity chromatography resin (Bio-Rad Laboratories, Inc., Hercules, CA) and to a Ni-iminodiacetic acid resin (Purolite Inc., Bala Cynwyd, PA), respectively.^12^ We herein refer to the Ni-nitrilotriacetic acid immobilized metal affinity chromatography resin as an acrylamide-based (AC) resin and the Ni-iminodiacetic acid resin as a methacrylate-based (ME) resin. The enzyme immobilization process was carried out using 8.0 g of the lyophilized PanK first being dissolved in immobilization buffer (50 mM sodium phosphate, 500 mM NaCl, 15 mM imidazole, pH 8.0) in a temperature controlled glass reaction vessel with overhead agitation. 50 mL of resin solids was initially washed to remove the storage solution. Resin was charged to a filter funnel, washed with 10 bed volumes (500 mL) of the immobilization buffer, and dried into a wet cake under vacuum. The cake was resuspended with 250 mL of immobilization buffer and transferred to a reaction vessel containing the dissolved enzyme. The mixture was held at 20 °C and stirred with overhead agitation at 300 rpm for 5 h. The mixture was filtered and washed four times with immobilization buffer (5 bed volumes per wash, 250 mL), holding for 15 min at each wash. Four additional washes with 50 mM Bis-Tris at pH 8 (5 bed volumes per wash, 250 mL) were similarly performed. The resin was dried to a wet cake over gentle vacuum and residual water removed by lyophilization.^36^ Prior to analysis, the immobilized enzyme system was placed on glass slides. To investigate the interior of immobilized enzyme beads, cross-sectional representations of PanK immobilized to both resins were prepared using a Leica EM UC7 ultramicrotome (Leica Microsystems, Wetzlar, Germany), in which the resin beads containing PanK were affixed to a glass slide via glue prior to the application of the microtome.

### 2.2 Raman Hyperspectral Imaging, Collection of Raman Reference Spectra, and Spectral Preprocessing Methods

All Raman hyperspectral imaging data was collected using an inVia Raman spectrometer (Renishaw, Wotton-under-Edge, United Kingdom) coupled with a DM2700 microscope (Leica Microsystems). Both 532 and 785 nm laser excitation sources were employed to access the optimal excitation wavelength for Raman hyperspectral imaging. For PanK immobilized to acrylamide-based (AC) resin, three Raman hyperspectral imaging data sets were acquired—the first data set contains 3339 total spectra collected from a 53 × 63 grid using 785 nm excitation; the second data set contains 3660 total spectra collected from a 60 × 61 grid using 532 nm excitation; the third data set contains 868 total spectra collected from a 28 × 31 grid using 785 nm excitation. For PanK immobilized to methacrylate-based (ME) resin, two Raman hyperspectral imaging data sets were acquired—the first data set contains 3192 total spectra collected from a 57 × 56 grid using 785 nm excitation; the second data set contains 10302 total spectra collected from a 102 × 101 grid using 532 nm excitation. The step size ranged from 1 to 2.5 μm in both the *x* and *y* directions. Each Raman spectrum was acquired using a 5 s of integration time, and the laser power was maintained at 25 mW.^37^

To establish a reference spectral library for all species used within the enzyme immobilization process, Raman microscopy was employed to analyze reference materials of each individual chemical species—lyophilized powder of PanK, AC resin, ME resin, Bis-Tris, glass substrate, glue, sodium phosphate (both solid and aqueous), sodium chloride, imidazole (both solid and aqueous), and propionic acid. Reference Raman spectra data collection was performed using a Renishaw inVia Raman spectrometer using both 532 and 785 nm excitation. A laser power of 25 mW was used with an integration time of 15 s. For PanK, a photobleaching step of 10 s was performed, followed by a 90 s accumulation period using 2.5 mW laser power.

All spectral preprocessing methods were applied using Python 3.7 with Anaconda Navigator and Jupiter Notebook. Prior to NMF analysis, an asymmetric least-squares baseline correction (adapted from SciPy) followed by a Savitzky–Golay filter (window size of 11, polynomial order of 3) was applied to all acquired Raman hyperspectral imaging data.^38^ Spectral normalization was also applied prior to determination of spectral similarity via scaling the minimum and maximum spectral intensity between zero and one, respectively.

### 2.3 Non-negative Matrix Factorization (NMF) Analysis

After application of spectral preprocessing methods, Anaconda Navigator with Jupiter Notebook was utilized to perform all analyses within Python 3.7. Application of NMF was performed using algorithms derived from scikit-learn. A detailed description of NMF, including mathematical relationships, algorithms, operational information, additional literature, comparison to other relevant machine learning methods, and extensive discussion of the technology, is available within the Supporting Information (sections S2 and S3). In short, multicomponent decomposition analysis of the obtained Raman hyperspectral imaging data sets via NMF was accomplished with default hyperparameters. NMF factorizes the experimental data matrix, *A*, into the product of two lower rank matrixes, *W* and *S*, according to *eq*1, where *E* represents the error unexplained by the bilinear model:
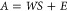
1Finding a bilinear model that satisfies *eq*1 requires definition of the cost function that describes the distance between *A* and *WS*. The cost function is optimized under a non-negativity constraint. In this study, the factors *W* and *S* were chosen to minimize the root-mean-squared residual between *A* and *WS*. This minimization is defined as follows, which utilizes a Frobenius norm:
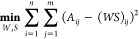
2To evaluate the performance and robustness of the NMF algorithm, relative reconstruction error (RRE) was employed as a metric to describe the error between original data, *A*, and the reconstructed matrix, *WS*.^39,40^ Calculation of RRE is described as follows using eq 3:
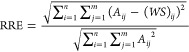
3

### 2.4 Similarity Score Determination

Quantitative determination of the similarity between NMF-resolved Raman spectra and Raman spectra of reference materials was accomplished using the cosine similarity metric, described in eq 4, as follows:
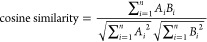
4Using this approach, the similarity of the NMF-resolved Raman spectra and the spectra of a given reference material can be calculated based on the cosine distance of their normalized spectra, where *A* and *B* represent the normalized spectral vector of the reference material and the normalized spectral vector of the NMF-resolved target, respectively.^41^ Intensity values from each spectrum are non-negative, so this similarity score will range between zero and one. The closer the similarity score value is to one, the more similar the reference spectrum is to the target data.

## 3 Results and Discussion

### 3.1 Raman Spectra of Reference Materials

**Figure 1:**
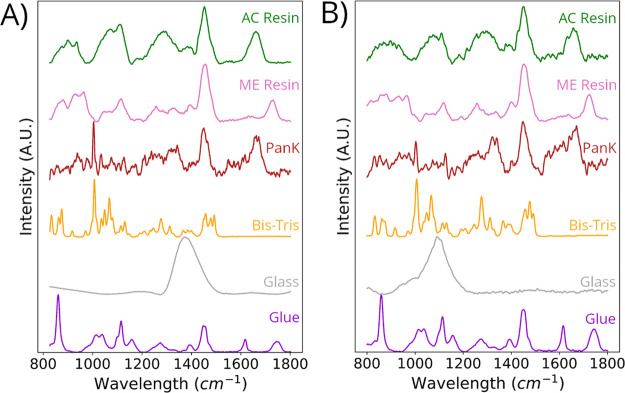
Representative Raman spectra obtained from reference materials of the key components used during the enzyme immobilization process. Spectra were collected under (A) 785 nm and (B) 532 nm excitation. The six species analyzed, listed from top to bottom, are acrylamide-based (AC) resin, methacrylate-based (ME) resin, pantothenate kinase (PanK), Bis-Tris, glass substrate, and adhesive glue.

Raman spectra were collected from 13 total reference materials to establish a reference spectral library for distinguishing all possible species used within the enzyme immobilization process (Figure S1). Reference Raman spectra were collected using both 532 and 785 nm excitation wavelengths. Selected Raman spectra acquired at both 532 and 785 nm excitation for species imperative to the enzyme immobilization process, including PanK, AC resin, ME resin, Bis-Tris, glass substrate, and adhesive glue, are displayed (Figure 1). In addition to the enzyme and inert microporous supports used for immobilization, there are three additional species of interest in this study—Bis-Tris, which is used as a final washing component during enzyme immobilization; glass substrate, which is used to hold the resin beads in place during analysis; and adhesive glue, which is exclusively utilized for samples that underwent microtome preparation prior to analysis.

Evaluation of the Raman spectra of the PanK reference material reveals a pronounced Raman band at 1003 cm^–1^, which can be attributed to phenylalanine. Notably, this band exhibits greater intensity under 785 nm excitation as compared to that of 532 nm excitation. The reference Raman spectrum of PanK displays shared bands with both resins originating from deformation of C–H bonds at 1454 cm^–1^. Additionally, a shared Raman band between PanK and AC resin at 1662 cm^–1^ is observed, indicative of the amide I region. Similarly, the reference Raman spectra of PanK overlap with that of Bis-Tris in the regions of 1003 and 1454 cm^–1^. Under 532 nm excitation, distinct variations in the reference Raman spectra can be observed across several reference species analyzed herein, especially with respect to PanK and Bis-Tris band intensities and band shifting for glass substrate. The characteristic Raman band for glass is at ∼1100 cm^–1^ using 532 nm excitation, while for 785 nm excitation, this band shifts to ∼1400 cm^–1^. The spectrum of glue does not greatly change between 532 and 785 nm excitation.^36^

### 3.2 Novel Approach for Determining Optimal NMF Models for Raman Hyperspectral Imaging Analysis

Due to significant overlap of Raman bands between enzyme and resins (Figure 1), relying solely on a specific Raman band from the analyte of interest to spatially resolve the hyperspectral imaging data would not yield a representative or accurate analysis. As such, NMF analysis—an unsupervised machine learning strategy—was conducted for decomposition of the hyperspectral imaging data to resolve individual, pure components within the mixture both spectrally and spatially.

Selecting the appropriate number of components for the NMF model construction remains a significantly challenging task. Selection using too few components will frequently fail to identify all species present, while selection using too many components will overfit the model. To address this issue, we propose a novel approach for the NMF analysis itself. Specifically, our approach involves constructing NMF models across a range of component numbers and quantitatively comparing the resulting NMF-resolved spectra to those of reference materials to select the optimal NMF model. In this new approach, we utilize three distinct quantifiable criteria to choose the optimal NMF model in a data-driven manner. First, the NMF model must accurately resolve all of the species present. Second, the NMF model must exhibit the highest spectral similarity score between the NMF-resolved spectra and that of reference materials. Third, the NMF model must demonstrate an alignment between the NMF-resolved spatial distributions and the information derived from the optical image.

**Figure 2:**
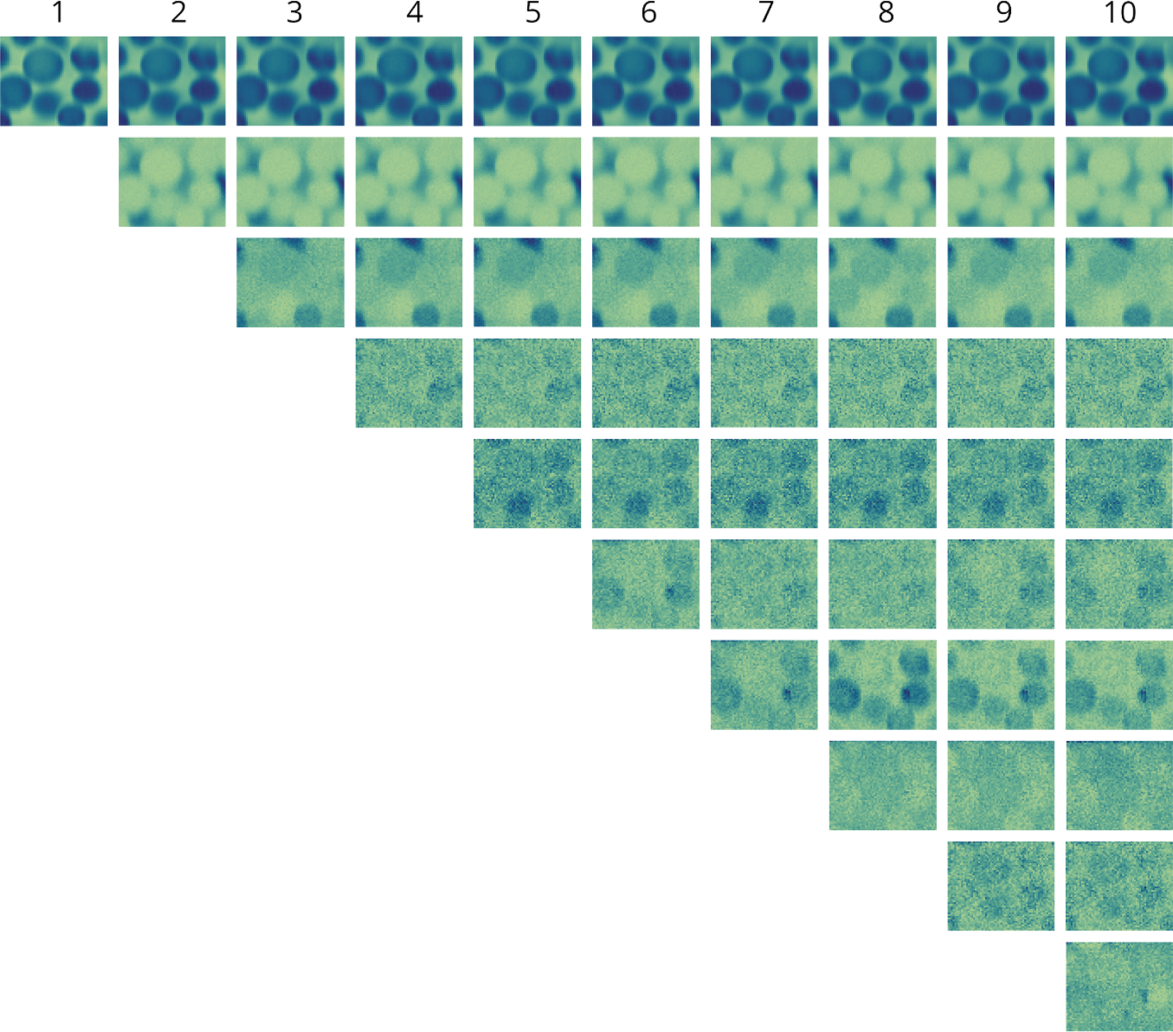
NMF-resolved concentration profiles formed into chemical images for PanK immobilized onto an acrylamide-based (AC) resin under 785 nm excitation. NMF models were constructed with varying numbers of components ranging from one to ten, in which the number at the top of each column represents the number of components used to construct the independent NMF model. Each column displays the chemical images resulting from the NMF analysis using the respective number of components.

**Figure 3:**
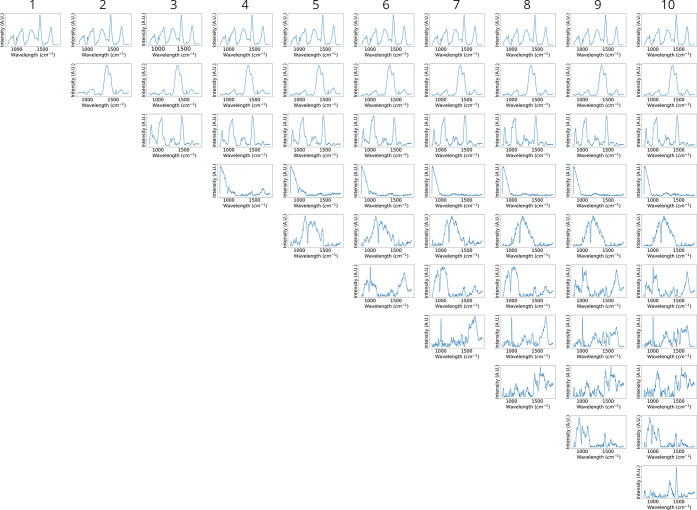
NMF-resolved Raman spectra for PanK immobilized onto an acrylamide-based (AC) resin under 785 nm excitation. NMF models were constructed with varying numbers of components ranging from one to ten, in which the number at the top of each column represents the number of components used to construct the independent NMF model. Each column displays the NMF-resolved Raman spectra resulting from the NMF analysis using the respective number of components.

Using our new NMF approach, we have analyzed five distinct Raman hyperspectral imaging data sets that span PanK immobilized to two distinct resins—both AC and ME—across unaltered immobilized systems as well as systems that have been cross-sectioned prior to analysis. In this manner, we offer a comprehensive and thorough evaluation of our proposed methodology for optimal NMF analysis. Full results for selecting the optimal NMF model using our new quantitative approach are displayed for PanK immobilized onto AC resin under 785 nm excitation (Figures 2 and 3). Full results for all other Raman hyperspectral imaging data sets are available in the Supporting Information—PanK immobilized onto the AC resin under 532 nm excitation (Figure S3 and S4), PanK immobilized onto ME resin under 785 nm excitation (Figure S5 and S6), PanK immobilized onto ME resin under 532 nm excitation (Figure S7 and S8), and microtomed PanK immobilized onto AC resin under 785 nm excitation wavelength (Figure S9 and S10).

For each hyperspectral data set, application of our new NMF approach results in ten independent models that each contain resolved concentration profiles and corresponding spectra. Comprehensive visualization of these results (i.e., Figures 2 and 3) enables a clear observation of the impact that increasing the number of components has on the NMF analysis. For NMF applied to Raman hyperspectral imaging of PanK immobilized to AC resin under 785 nm excitation, the concentration profiles (Figure 2) are reconstructed into chemical images to elucidate spatial distributions. Similarly, the corresponding NMF-resolved spectra (Figure 3) provide molecular information for component identification. This spatial (Figure 2) and spectral (Figure 3) information demonstrates a clear result—systematic progression from lower numbers of components failing to resolve all species while higher numbers of components potentially beginning to overfit the model.

**Figure 4:**
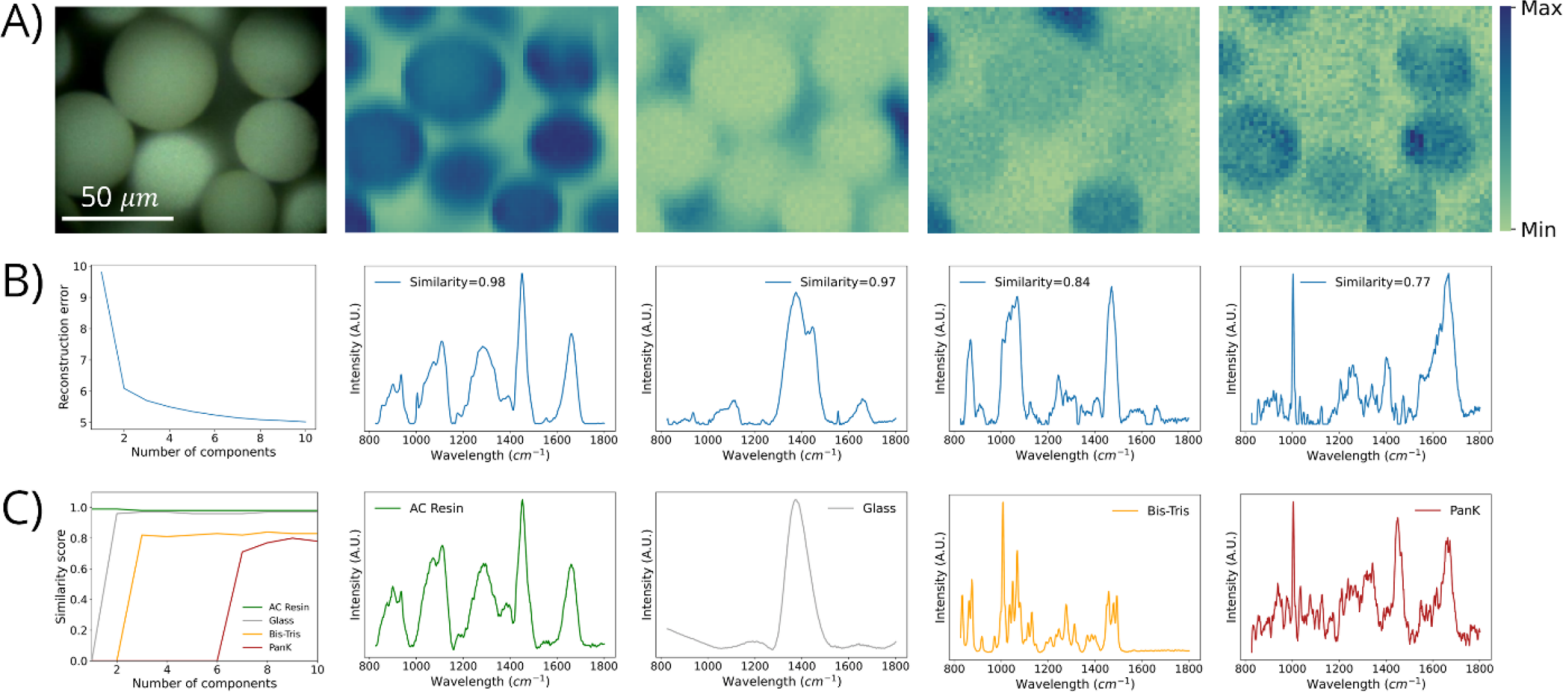
Summary of results for NMF with Raman hyperspectral imaging of PanK immobilized to acrylamide-based (AC) resin by using 785 nm excitation. (A) Optical image and NMF-resolved chemical images of AC resin, glass substrate, Bis-Tris, and PanK (left to right). (B) Relative reconstruction error (RRE) plot and NMF-resolved Raman spectra that correspond to AC resin, glass substrate, Bis-Tris, and PanK (left to right). For the RRE plot, the *x*-axis represents the number of components used to construct independent NMF models and the *y*-axis represents the relative reconstruction error. For the NMF-resolved Raman spectra, the cosine spectral similarity score is displayed within each spectra. (C) Spectral similarity plot with increasing number of NMF components and Raman spectra of reference materials—AC resin, glass substrate, Bis-Tris, and PanK (left to right).

Our results suggest that traditional NMF approaches fail to adequately analyze the hyperspectral imaging data herein, yet our novel NMF methodology indeed successfully resolves, both spatially and spectrally, all of the species present. If we were to adopt conventional methodologies for selecting the NMF model, we would typically refer to the relative reconstruction error (RRE) and assess the performance of the NMF model in this manner. In the case of PanK immobilized to AC resin under 785 nm excitation, the RRE plots (Figure 4B) suggest that the optimal NMF model would entail either two or three components. Using this typical approach, our optimal NMF model would resolve only AC resin and glass substrate or, at best, AC resin, glass substrate, and Bis-Tris. Identification of PanK, a species of utmost importance, would thus not be accomplished if conventional NMF approaches were used. Using our novel approach, however, PanK is indeed resolved if seven or more components are utilized. Moreover, an eight component NMF model yields the highest spectral similarity and achieves the best agreement between the NMF-resolved chemical distributions and the optical image.

For this data set, an eight component NMF model is selected as optimal, in which the complete criteria for our novel NMF methodology are achieved. Specifically, this model is selected as optimal due to the following measures—first, the model successfully resolved all four species present (i.e., PanK, AC resin, glass substate, and Bis-Tris); second, the model displayed the highest spectral similarity score between NMF-resolved spectra with that of reference material; and third, the model displayed strong agreement between NMF-resolved chemical distributions with the optical image.

Application of the optimal NMF model approach presented herein was further demonstrated across the four additional Raman hyperspectral imaging data sets studied in this work. Our observations reveal that NMF analysis with conventional component numbers often fails to adequately resolve all species present, especially difficult to identify or lower abundance species. Our proposed NMF approach, however, is able to spatially and spectrally resolve all species present within the hyperspectral imaging data sets analyzed herein and, as such, can overcome this challenging task at which conventional methods frequently fail.

### 3.3 Cosine Similarity Score

In order to determine the optimal NMF model using the approach outlined herein, a quantitative evaluation of the similarity between NMF-resolved spectra and reference Raman spectra is needed. This is primarily due to two main reasons. First, the degree of similarity between NMF-resolved spectra and those of reference materials provides chemical and molecular identification. Second, the highest spectral similarity between NMF-resolved and reference spectra is a key criterion for optimal NMF model selection. In this study, we utilized the cosine similarity score, as defined in section section 2.4, as a quantitative metric to assess the agreement between NMF-resolved spectra and reference Raman spectra. The cosine similarity score provides a quantitative measure of similarity between the two spectra, with a value closer to one indicating a stronger resemblance.

The comprehensive cosine similarity score results for PanK immobilized in AC resin under 785 nm excitation are displayed (Table 1). The results for the four additional data sets analyzed in this work are available in the Supporting Information. This includes comprehensive cosine similarity score results for PanK immobilized to ME resin under 785 nm excitation (Table S2), PanK immobilized to ME resin under 532 nm excitation (Table S3), PanK immobilized to AC resin under 532 nm excitation (Table S4), and micromed PanK immobilized to AC resin under 785 nm excitation (Table S5). In total, over 220 spectral similarity scores were elucidated for each of the five hyperspectral imaging data sets studied herein (i.e., four reference Raman spectra compared to each NMF-resolved spectra across ten NMF models, ranging from one to ten components). This comprehensive analysis allows for a thorough evaluation of our optimal NMF model selection approach and highlights the identification of relevant species via our proposed NMF analysis.

**Table 1:** Results of Spectral Similarity between NMF-Resolved Spectra and Reference Materials Using Hyperspectral Imaging (785 nm Excitation) of PanK Immobilized to Acrylamide (AC) Resin^a^

		Spectral Similarity			
NMF Model Number	Component Number	Glass	Acrylamide Resin	PanK	Bis-Tris
NMF model 1	1	0.48	0.99	0.90	0.62
NMF model 2	1	0.44	0.99	0.89	0.62
	2	0.96	0.66	0.66	0.40
NMF model 3	1	0.44	0.98	0.89	0.60
	2	0.97	0.60	0.62	0.31
	3	0.27	0.80	0.65	0.82
NMF model 4	1	0.44	0.98	0.90	0.60
	2	0.97	0.62	0.63	0.34
	3	0.26	0.81	0.65	0.81
	4	0.20	0.44	0.44	0.40
NMF model 5	1	0.44	0.98	0.90	0.60
	2	0.96	0.62	0.64	0.34
	3	0.27	0.81	0.65	0.82
	4	0.20	0.40	0.38	0.43
	5	0.44	0.77	0.67	0.50
NMF model 6	1	0.44	0.98	0.89	0.59
	2	0.96	0.61	0.62	0.33
	3	0.24	0.76	0.61	0.83
	4	0.21	0.34	0.31	0.37
	5	0.39	0.75	0.66	0.54
	6	0.20	0.60	0.73	0.43
NMF model 7	1	0.44	0.98	0.89	0.59
	2	0.96	0.62	0.61	0.33
	3	0.24	0.74	0.61	0.82
	4	0.18	0.31	0.28	0.33
	5	0.36	0.66	0.62	0.43
	6	0.17	0.68	0.59	0.64
	7	0.23	0.45	0.71	0.26
NMF model 8*	1	0.44	***0.98***	0.88	0.59
	2	***0.97***	0.61	0.60	0.33
	3	0.28	0.72	0.61	***0.84***
	4	0.17	0.30	0.28	0.33
	5	0.37	0.62	0.58	0.45
	6	0.16	0.68	0.59	0.64
	7	0.31	0.55	***0.77***	0.30
	8	0.23	0.57	0.69	0.35
NMF model 9	1	0.44	0.98	0.88	0.59
	2	0.97	0.60	0.60	0.32
	3	0.25	0.73	0.61	0.83
	4	0.17	0.30	0.27	0.33
	5	0.38	0.63	0.58	0.47
	6	0.22	0.68	0.72	0.60
	7	0.36	0.58	0.80	0.41
	8	0.26	0.62	0.72	0.39
	9	0.20	0.60	0.54	0.48
NMF model 10	1	0.44	0.98	0.88	0.59
	2	0.97	0.60	0.59	0.32
	3	0.24	0.74	0.60	0.83
	4	0.18	0.32	0.28	0.34
	5	0.39	0.63	0.58	0.47
	6	0.25	0.68	0.72	0.60
	7	0.31	0.57	0.78	0.40
	8	0.22	0.65	0.69	0.42
	9	0.21	0.58	0.55	0.46
	10	0.57	0.63	0.71	0.44

a Note: The optimal NMF model is denoted by *. The spectral similarity score for each final resolved species is bolded and italicized for ease of visualization.

The spectral similarity scores for PanK immobilized to AC resin under 785 nm excitation (Table 1) exhibit strong agreement with the NMF-resolved spectral cascade (Figure 3). As the number of components in our NMF model increases, it becomes evident that the first component consistently corresponds to AC resin. This is supported by cosine similarity scores exceeding 0.99 when comparing NMF-resolved spectra of the first component across models with the AC resin reference spectrum. Similarly, the second component is consistently identified as the glass substrate, with cosine similarity scores greater than 0.96 observed when comparing NMF-resolved spectra across all models with the glass substrate reference spectrum. The third component is identified as Bis-Tris, as indicated by cosine similarity scores greater than 0.81 when comparing NMF models constructed using three or more components. Moreover, a three component model represents the first time Bis-Tris is resolved using NMF analysis. These findings confirm the presence and successful resolution of three species—AC resin, glass substrate, and Bis-Tris—via NMF analysis.

Using a traditional NMF analysis approach, the optimal NMF model would be constructed with two or a maximum of three components and thus fail to resolve PanK for this data set. In contrast, our new NMF methodology reveals that PanK is identified and successfully resolved only if seven components are utilized for NMF analysis, resulting in a spectral similarity of 0.71 (Table 1). The optimal NMF model for this data set was determined to be an eight component model, as this NMF model yielded a higher spectral similarity of 0.77 with PanK and demonstrated good agreement between the NMF-resolved chemical distributions and the optical information. Increasing the number of components beyond eight did not significantly improve the similarity score of PanK. It is noteworthy that this eight component NMF model is further considered optimal, as it provides high spectral similarities under the most parsimonious conditions, given that PanK was initially resolved using seven components.

We also observe high statistical stability for the four identified species in this data set using our NMF analysis, in which the cosine similarity scores are both high and unfluctuating once a particular species is resolved. For instance, in this data set, AC resin is resolved using low NMF component numbers and demonstrates a stable cosine similarity score of ≥0.98 across constructed NMF models. Similar statistical stability is demonstrated for glass substrate, which displays consistent cosine similarity scores of ≥0.96 across the relevant NMF models. Finally, both Bis-Tris and PanK also have high statistical stability with respect to the determined cosine similarity scores, in which similarities of ≥0.81 and ≥0.71, respectively, with low variance are observed. Clearly, once a given chemical species is resolved using an NMF model, statistical stability in the cosine similarity scores is observed.

### 3.4 NMF with Hyperspectral Imaging of PanK Immobilized to Acrylamide (AC) Resin

Application of our novel NMF approach to hyperspectral imaging data collected under 785 nm excitation of PanK immobilized to AC resin (Figure 4) resulted in spatial and spectral resolution of all individual chemical species present within this immobilized enzyme system. Four distinct species were resolved—PanK, AC resin, Bis-Tris, and glass substrate. Resolution of these four distinct species was accomplished by first determining the optimal NMF model, as described in section 3.3. In particular, for this AC resin-based data set, the NMF-resolved and reference spectra exhibit excellent agreement, displaying a high spectral similarity of 0.98. The resolved concentration profile for AC resin demonstrated strong correspondence to the optical image (Figure 4), especially with respect to the resin beads and their edges. Each resin bead observed in the optical image was reflected in the NMF-resolved AC resin chemical image. The totality of these results, including the NMF-resolved concentration profiles, the alignment of chemical maps with optimal images, and the cosine similarity scores, provides clear evidence that AC resin was successfully resolved by using this methodology.

The NMF-resolved spectrum for the glass substrate demonstrated excellent agreement with the reference spectrum, displaying a high spectral similarity of 0.97. The resolved concentration profile from the optimal NMF model clearly indicates the presence of a glass substrate outside of the resin beads, as expected. This observation aligns with the locations observed in the optical image (Figure 4), wherein the glass is visible in the regions not occupied by the resin beads. The NMF-resolved spatial distribution of the glass substrate also allows for further validation of resin bead positions.

For Bis-Tris, the NMF-resolved spectrum and the reference spectrum demonstrated strong agreement with a high spectral similarity 0.84. The NMF-resolved spectrum exhibited prominent Raman bands in the range of 800–900 cm^–1^, 1000–1100 cm^–1^, and 1400–1500 cm^–1^ that are characteristic of Bis-Tris. The resolved concentration profile from the optimal NMF model revealed that Bis-Tris was observed only in a subset of the resin beads. This observation is consistent with Bis-Tris being used as a final washing step during the immobilization process, indicating its residual presence after washing.

Regarding PanK, the NMF-resolved spectrum and the reference spectrum displayed great agreement, showing a high spectral similarity of 0.77. The resolved concentration profile from the optimal NMF model elucidates that PanK was also present in only a subset of the resin beads. Interestingly, the distribution of PanK correlated inversely with the distribution of Bis-Tris, with higher PanK localities corresponding to lower Bis-Tris and *vice versa*. This finding is consistent with the role of Bis-Tris as a final washing step during the immobilization process. Furthermore, this result suggests that choosing a different final washing step or using a lower concentration of Bis-Tris may result in higher PanK coverage during the immobilization process. The spatial distributions of PanK and resin are found to be colocated, indicating that PanK is indeed affixed to the resin during the immobilization process.

The results of the optical image and NMF-resolved spatial profiles (Figure 4) highlight the distinct ability of this methodology to simultaneously elucidate the chemical distributions of highly overlapped, difficult to unmix species. The RRE plots and NMF-resolved Raman spectra (Figure 4) demonstrate clear resolution of more species than predicted by using RRE approaches. The Raman spectrum of each reference material is placed beneath the NMF-resolved spectrum (Figure 4) for ease of comparison. The NMF-resolved spectra are all in great agreement with their respective reference Raman spectra, which is further evidenced by the spectral similarities displayed across AC resin, glass substrate, Bis-Tris, and PanK. Finally, the plot of the similarity score with increasing component numbers (Figure 4C) shows the clear progression of initial resolution of resin, then glass, and then Bis-Tris with one, two, and three component NMF models, respectively, with a final resolution of PanK for the first time starting with seven components while ultimately stabilizing and providing a final NMF model of eight components. High statistical stability of the cosine similarity scores is observed for all four resolved species, as evidenced by the relatively constant and unfluctuating spectral similarities we observed (Figure 4C). Further, when AC resin, glass, Bis-Tris, and PanK are initially resolved, we observe a steep increase in spectral similarity as expected (i.e., the transition from not identified to identified), and thereafter, stable cosine similarity scores are shown. The similarity scores of NMF-resolved spectra with additional reference species not present within this hyperspectral image, including sodium phosphate, imidazole, and propionic acid, were calculated as a control (Figure S11), in which all observed similarity scores were less than 0.5 and thus allow for a threshold for straightforward evaluation. Additional discussion of these spectral similarity scores is available in the Supporting Information (section S7).

Results from application of NMF to hyperspectral imaging data collected on PanK immobilized onto AC resin using 532 nm excitation (Figures S2, S3, and S4; Table S4) further demonstrate the ability of this methodology to resolve PanK and AC resin. NMF-resolved concentration profiles (Figure S3) and corresponding NMF-resolved Raman spectra (Figure S4) for PanK immobilized to AC resin under 532 nm excitation were generated across ten independent NMF models by varying the number components from one to ten. Following this approach, the resulting spatial and spectral resolutions are clear and visualized via the spectral and distribution cascades (Figures S3 and S4). These results emphasize the importance of optimally selecting the number of components for the NMF model not only based on the evaluation matrix, such as relative reconstruction error, but based on the new criteria that we have described in our methodology herein—resolution of all chemical species present, high spectral similarity scores, and agreement of chemical distributions with optical imaging. Detailed discussion of NMF analysis applied to this data set is available in the Supporting Information (section S2).

### 3.5 NMF with Hyperspectral Imaging of PanK immobilized to Methacrylate (ME) Resin

**Figure 5:**
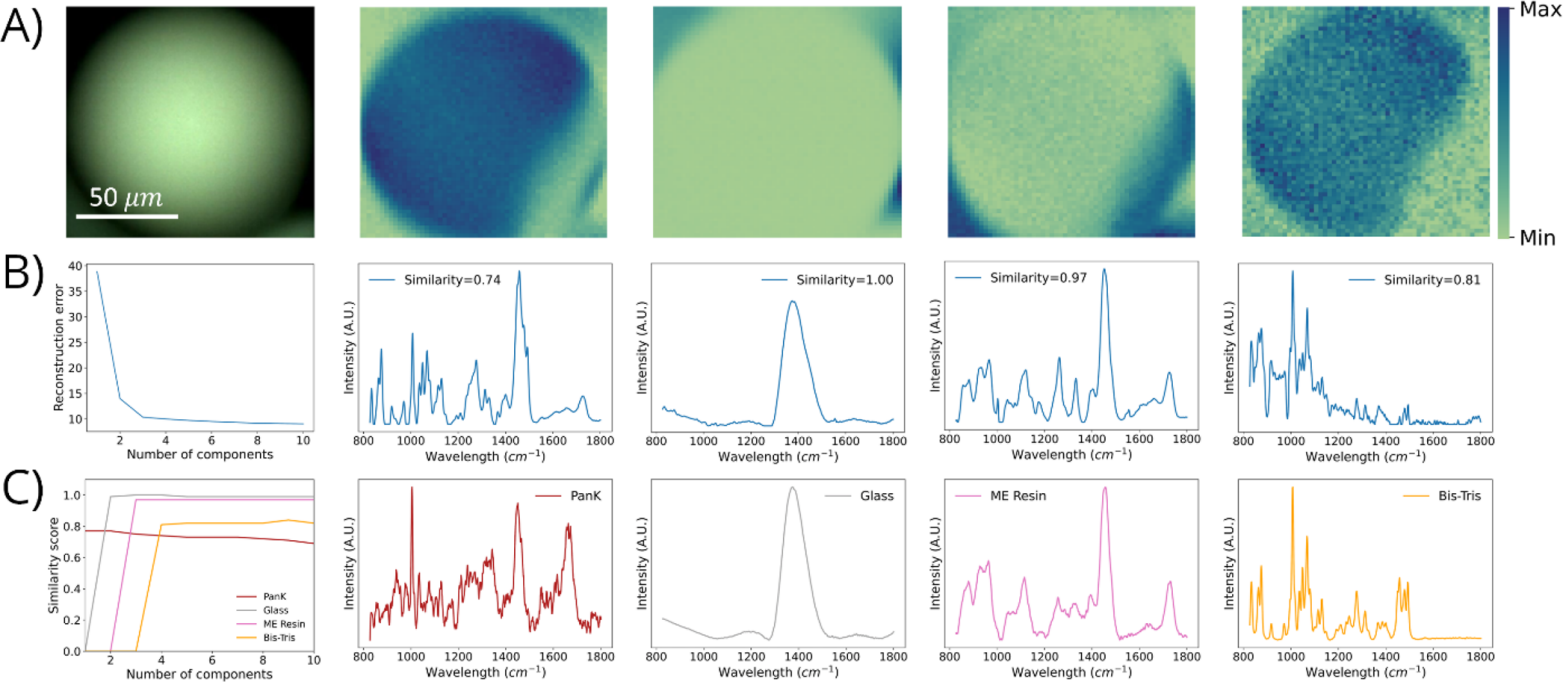
Summary of the Results for NMF with Raman hyperspectral imaging of PanK immobilized to methacrylate-based (ME) resin using 785 nm excitation. (A) Optical image and NMF-resolved chemical images of PanK, glass substrate, ME resin, and Bis-Tris (left to right). (B) Relative reconstruction error (RRE) plot and NMF-resolved Raman spectra that correspond to PanK, glass substrate, ME resin, and Bis-Tris (left to right). For the RRE plot, the *x*-axis represents the number of components used to construct independent NMF models and the *y*-axis represents the relative reconstruction error. For the NMF-resolved Raman spectra, the cosine spectral similarity score is displayed within each spectra. (C) Spectral similarity plot with increasing number of NMF components and Raman spectra of reference materials—PanK, glass substrate, ME resin, and Bis-Tris (left to right).

Results of NMF analysis applied to Raman hyperspectral imaging of PanK immobilized onto the ME resin using 785 nm excitation (Figure 5) illustrate the spatial and spectral resolution of four distinct species—PanK, glass substrate, ME resin, and Bis-Tris. The optical image depicts a single resin bead of approximately 100 μm in diameter (Figure 5A), in which a total of 3192 Raman spectra were collected in a 56 × 57 nm grid to form this hyperspectral imaging data set.

To select the optimal NMF model for deconvolution, ten independent NMF models were constructed that ranged in number of components from one to ten. The detailed NMF-resolved chemical images (Figure S5) and corresponding NMF-resolved Raman spectral cascade (Figure S6) for all ten NMF models are available in the Supporting Information. Based on traditional RRE-based approaches to selecting the best NMF model, this data set would suggest a two or maximum of three components. However, our novel NMF methodology reveals that this traditional approach fails to successfully resolve all species present. A one component NMF model yields resolution of only PanK. A two component NMF model yields resolution of PanK and glass substrate. A three component NMF model yields resolution of PanK, glass substrate, and ME resin. A four component NMF model yields resolution of PanK, glass substrate, ME resin, and Bis-Tris. NMF models constructed with more than four components did not yield resolution of any additional chemical species and consistently resolved PanK, glass substrate, ME resin, and Bis-Tris. Therefore, to successfully elucidate all the species present, our optimal NMF methodology requires at least a four component model.

Results for spectral similarities between the NMF-resolved spectra and the reference spectra for this data set (Table S2) demonstrate that the highest similarity was achieved using a four component NMF model. Using a four component NMF model, glass substrate displayed a spectral similarity of 1.00, ME resin displayed a spectral similarity of 0.97, PanK displayed a spectral similarity of 0.74, and Bis-Tris displayed a spectral similarity of 0.81. Further, the spectral similarity scores for these four components demonstrated clear statistical stability, as evidenced by the low variance and consistently high spectral similarities observed (Figure 5C) across ME resin, glass substrate, PanK, and Bis-Tris in NMF models that resolved these species. Increasing the component numbers of NMF models did not result in any significant increases in the spectral similarities across the four resolved species. This observation allows for the most parsimonious model to be selected for this data set as optimal. The chemical distributions using this optimal four component model were in good agreement with the optical image. As such, a four component NMF model was determined to be the optimal model for this data set.

For the optimal NMF model for Raman hyperspectral imaging of PanK immobilized to ME resin using 785 nm excitation (Figure 5), four distinct chemical species were spatially and spectrally resolved—PanK, glass substrate, ME resin, and Bis-Tris. As described previously, the RRE results (Figure 5B) demonstrate that a traditional NMF analysis would be applied using only two or possible three components; however, this RRE-based approach would not yield resolution for all present chemical species. Our optimal NMF model selected using four components was able to successfully resolve all species present, demonstrate high spectral similarity with reference spectra, and was in agreement with NMF-resolved chemical distributions. Adding additional NMF components for this data set did not yield any significant improvements in the RRE or in the spectral similarities, and consequently, this further demonstrates the selection of this NMF model as most optimal.

The NMF-resolved chemical images (Figure 5A) and corresponding NMF-resolved Raman spectra (Figure 5B) highlight the clear spatial and spectral resolution of this data set using our proposed methodology. The cosine similarity score determination is summarized (Figure 5C), in which selection of the most optimal NMF model is further validated. The chemical imaging results indicate that the distribution of PanK within the resin beads is nonuniform, as evidenced by the concentration decrease observed for PanK at the lower right corner of the image, where the ME resin is located. The resolved Bis-Tris chemical distribution overlaps with that of PanK, particularly in areas where there is a high abundance of the enzyme, suggesting that residual Bis-Tris may remain after final washing step. While Bis-Tris can effectively remove any nonspecifically bound species from resin, it may leave residual Bis-Tris within the resin bead itself. The identification of residual Bis-Tris in the hyperspectral image is a critical finding, as this may impact the functionality of the immobilized enzyme. Given this result, modifying the process to identify a more appropriate washing solution to improve the enzyme immobilization and thus help increase biocatalytic performance may be warranted. The observed distribution of the ME resin further suggests the presence of Bis-Tris on the surface of the beads, given their complementary nature. The distribution of glass substrate is in great agreement with the optical image, in which the spatial locality of glass is exterior to the resin bead as expected. Finally, the NMF-resolved Raman spectra (Figure 5B) are in great agreement with the Raman spectra of reference material (Figure 5C), as shown by both the qualitative alignment and interpretation of the spectra (i.e., comparison of Figure 5B with Figure 5C) as well as the high spectral similarities observed.

**Figure 6:**
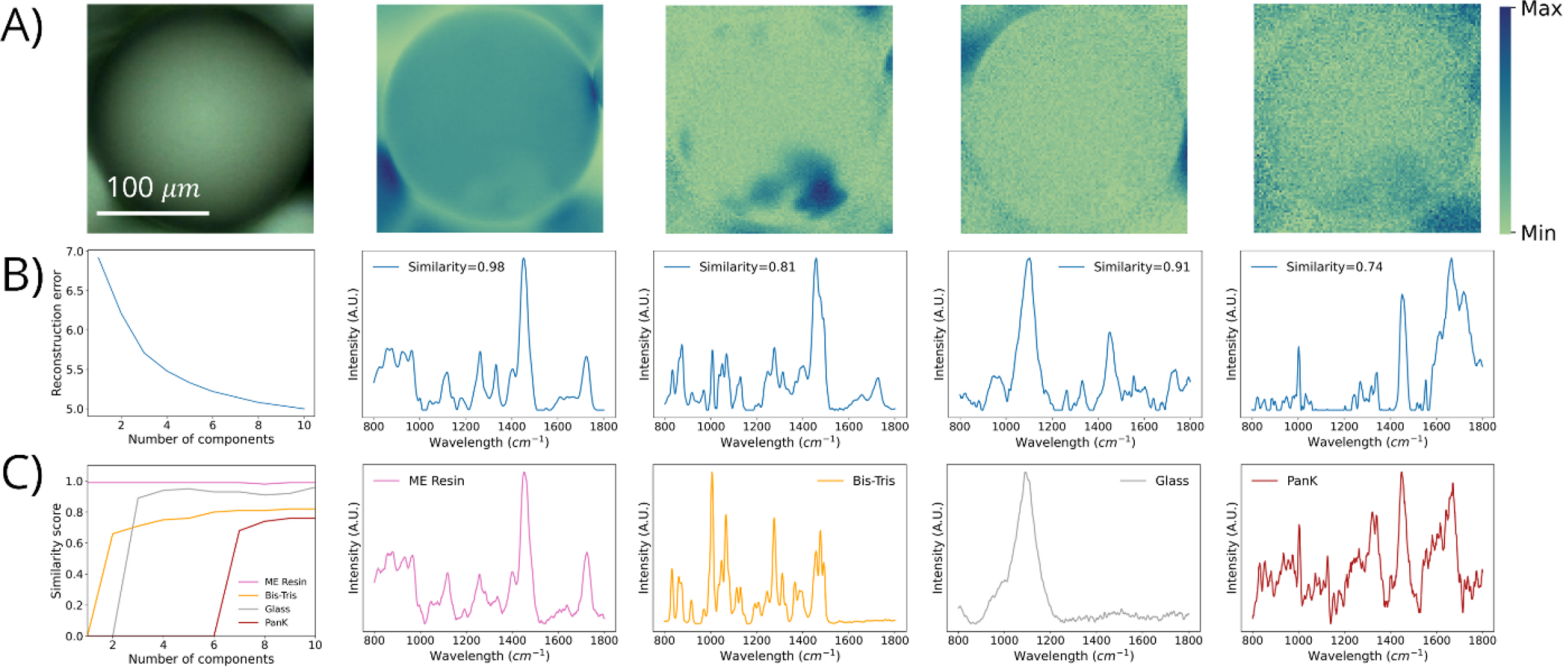
Summary of results for NMF with Raman hyperspectral imaging of PanK immobilized to a methacrylate-based (ME) resin using 532 nm excitation. (A) Optical image and NMF-resolved chemical images of ME resin, Bis-Tris, glass substrate, and PanK (left to right). (B) Relative reconstruction error (RRE) plot and NMF-resolved Raman spectra that correspond to ME resin, Bis-Tris, glass substrate, and PanK (left to right). For the RRE plot, the *x*-axis represents the number of components used to construct independent NMF models and the *y*-axis represents the relative reconstruction error. For the NMF-resolved Raman spectra, the cosine spectral similarity score is displayed within each spectra. (C) Spectral similarity plot with increasing number of NMF components and Raman spectra of reference materials—ME resin, Bis-Tris, glass substrate, and PanK (left to right).

Results of NMF with PanK immobilized to ME resin using 532 nm excitation (Figure 6) highlight the complete deconvolution of this complex hyperspectral image to yield four distinct species—ME resin, Bis-Tris, glass substrate, and PanK. The optical image (Figure 6A) shows a single resin bead of approximately 200 μm in diameter. To generate the hyperspectral imaging data set, a total of 10302 Raman spectra were collected in a 101 × 102 grid. Detailed results and discussion on the selection of the optimal number NMF model based on chemical images, Raman spectra, and similarity scores can be found in Supporting Information. In short, determination of the optimal NMF model for analysis of this hyperspectral image began with constructing ten independent NMF models ranging from one to ten components. For all ten independent NMF models, the full NMF-resolved chemical images (Figure S7) and corresponding NMF-resolved Raman spectra (Figure S7) are available in Supporting Information, allowing for straightforward visualization of all generated chemical distributions and resolved spectra on a per NMF model basis. Subsequent to building all NMF models, we evaluated the traditional RRE-based approach to selecting the best NMF model. Using this traditional RRE-based approach (Figure 6A), an NMF model with three components would most likely be selected. If this were the case, only ME resin and Bis-Tris would be resolved. As we describe herein, our novel NMF approach is able to spatially and spectrally resolve all four distinct chemical species, further demonstrating the usefulness of our new methodology. Specifically, for this data set, a one component NMF model yielded only ME resin; a two component model yielded ME resin and Bis-Tris; a six component model yielded ME resin, Bis-Tris, and glass substrate; and an eight component model yielded ME resin, Bis-Tris, glass substrate, and PanK. NMF models built using more than eight components yielded no additional chemical species present.

The optimal NMF analysis for resolution of all species within this data set was determined to be an eight component model. We observed that to resolve all species present (i.e., ME resin, Bis-Tris, glass substrate, and PanK), eight or more components were needed. Using our approach, selection of the optimal NMF model relies on determining the model that demonstrates the highest spectral similarities between NMF-resolved and reference spectra. Results for spectral similarities of NMF-resolved spectra compared to reference spectra (Table S3) demonstrate the highest spectral similarity was achieved using an eight component NMF model. Using this model, ME resin displayed a spectral similarity of 0.98, the glass substrate displayed a spectral similarity of 0.91, Bis-Tris displayed a spectral similarity of 0.81, and PanK displayed a spectral similarity of 0.74. Spectral similarity trends for these four components revealed statistical stability was achieved, as evidenced by the relative flat and unfluctuating trends observed for the resolved chemical species when plotting the similarity score relative to the number of components for NMF analysis (Figure 6C). Increasing the NMF component numbers above eight did not yield any significant increases in the spectral similarities across these four species. NMF-resolved chemical distributions for this model (Figure 6A) were in agreement with the optical image. Considering the totality of this information, the optimal NMF model was selected by using eight components. This model resolved all species present, displayed high spectral similarities of NMF-resolved spectra with reference spectra, and demonstrated chemical distributions that were in agreement with the respective optical information.

The NMF-resolved chemical images (Figure 6A) and corresponding NMF-resolved Raman spectra (Figure 6B) illustrate the spatial and spectral resolution of PanK immobilized to ME resin using 532 nm excitation using our proposed methodology. The cosine similarity scores (Figure 6C) confirm the selection of our NMF model as most optimal. The chemical distributions elucidated by NMF analysis indicate that the resin is observed throughout the entirety of the bead. The distribution of Bis-Tris shows a localized distribution on a limited portion of the resin bead. The distribution of PanK shows locations throughout the resin bead in a complementary fashion with Bis-Tris. The distribution of glass substrate shows glass is present outside of the resin bead in great agreement with the optical image. Based on these spatial distributions, we note that residual Bis-Tris is present and may impact enzymatic performance for biocatalysis. This observation allows for a potential focal point for process development efforts. The NMF-resolved Raman spectra (Figure 6B) are in agreement with the Raman spectra of the respective reference material (Figure 6C). This is evidenced by comparison and interpretation of the spectra and evaluation of the high cosine spectral similarities, which both suggest adequate spectral resolution of the four species observed.

### 3.6 NMF with Hyperspectral Imaging of Microtomed PanK Immobilized to Acrylamide (AC) Resin

**Figure 7:**
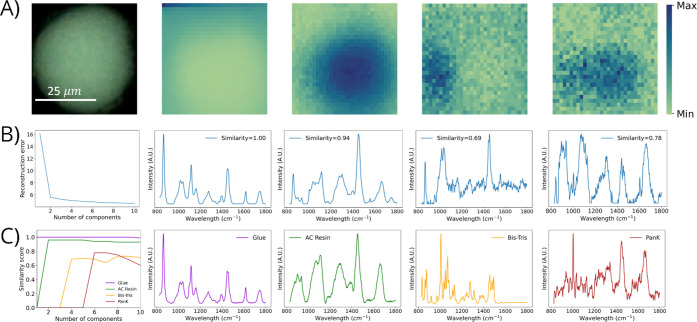
Summary of results for NMF with Raman hyperspectral imaging of micromed PanK immobilized to acrylamide-based (AC) resin by using 785 nm excitation. (A) Optical image and NMF-resolved chemical images of adhesive glue, AC resin, Bis-Tris, and PanK (left to right). (B) Relative reconstruction error (RRE) plot and NMF-resolved Raman spectra that correspond to adhesive glue, AC resin, Bis-Tris, and PanK (left to right). For the RRE plot, the *x*-axis represents the number of components used to construct independent NMF models and the *y*-axis represents the relative reconstruction error. For the NMF-resolved Raman spectra, the cosine spectral similarity score is displayed within each spectra. (C) Spectral similarity plot with increasing number of NMF components and Raman spectra of reference materials—adhesive glue, AC resin, Bis-Tris, and PanK.

Results of NMF with Raman hyperspectral imaging of microtomed PanK immobilized to AC resin using 785 nm excitation (Figure 7) allow for the analysis of a cross-sectional representation of immobilized enzyme systems. This immobilized enzyme system underwent sample preparation via microtome analysis prior to hyperspectral imaging data collection in order to provide a cross-section of the AC resin bead containing immobilized PanK. The optical image (Figure 7A) shows a flat, cross-sectioned resin bead that remains mostly circular in shape and is about 50 μm in diameter. To generate the hyperspectral imaging data set, a total of 868 Raman spectra were collected in a 28 × 31 grid.

Initially, traditional RRE-based approaches to selecting the best NMF model were assessed. Using a traditional RRE-based approach (Figure 7A), an NMF model using two components would be selected. A two component NMF model resolves only adhesive glue, which is needed to perform microtome sample preparation, and AC resin. Using our novel NMF methodology, however, four distinct species were resolved—adhesive glue, AC resin, Bis-Tris, and PanK. It is important to highlight that using traditional NMF modeling, two important chemical species would go unidentified and unresolved, yet using our new NMF methodology with optimal component selection, all chemical species present are successfully deconvoluted. For this microtomed immobilized enzyme system (Figure 7C and Table S5), a one component NMF model yielded only adhesive glue; a two component model yielded adhesive glue and AC resin; a four component model yielded adhesive glue, AC resin, and Bis-Tris; and a six component model yielded adhesive glue, AC resin, Bis-Tris, and PanK. NMF models built by using more than six components yielded no additional species present.

As outlined herein, our novel NMF approach relies on the first criteria for optimal model selection, the model must resolve all chemical species present. As such, this microtomed data set must select models containing at least six NMF components, as this was the first model to resolve all four chemical species—adhesive glue, AC resin, Bis-Tris, and PanK—present. Of the subset of models that resolves all species (i.e., NMF models with components ≥ six), determining the highest spectral similarities between NMF-resolved spectra and reference spectra is the next criterion for our NMF methodology. Results for spectral similarities (Table S5) demonstrate the highest similarity using a six component NMF model. Using this model, adhesive glue displayed a spectral similarity of 1.00, AC resin displayed a spectral similarity of 0.94, PanK displayed a spectral similarity of 0.78, and Bis-Tris displayed a spectral similarity of 0.69. Spectral similarity trends (Figure 7C) for these four species show relatively flat and unchanging behavior, suggesting the statistical stability of the spectral similarity scores for the resolved species. Further increasing NMF components did not yield any significant improvement in the spectral similarities across the four resolved species. NMF-resolved chemical distributions (Figure 7A) were in agreement with the optical image. The optimal model was thus selected using six NMF components due to this model resolving all species, having high spectral similarities of NMF-resolved spectra with reference spectra and demonstrating chemical distributions in agreement with optical information.

The NMF-resolved chemical images (Figure 7A) and corresponding NMF-resolved Raman spectra (Figure 7B) show that our proposed methodology can indeed analyze microtomed cross-sectional representations of the immobilized enzyme systems. Specifically, our proposed technology demonstrated complete spatial and spectral resolution of microtomed PanK immobilized to AC resin using 785 nm excitation (Figure 7), yielding spatial distributions and molecular identification of four distinct species—adhesive glue, AC resin, Bis-Tris, and PanK. The cosine similarity scores (Figure 7C) demonstrate agreement between reference spectra and NMF-resolved spectra. The chemical distributions elucidated by NMF show that we have now identified adhesive glue, which is used to fix the resin to the substrate for microtome sample preparation. The distribution of adhesive glue is primarily exterior to the micromolded resin bead. The distribution of AC resin is in agreement with the optical image and shows that the resin is intuitively located within the cross-sectioned bead. The distribution of Bis-Tris is highly localized within the image in a fashion complementary to that of PanK. Moreover, the distribution of PanK is observed within the bead, covering a substantial amount of the cross-sectioned area. The NMF-resolved Raman spectra (Figure 7B) are in agreement with the Raman spectra of the respective reference material (Figure 7C). Spectral resolution of these four species is further apparent via visual comparison of NMF-resolved spectra with reference spectra (compare Figure 7B with Figure 7C) along with the observed high cosine spectral similarities (Table S5).

## 4 Conclusions

Leveraging directed enzyme evolution for biocatalysis has resulted in tremendous advancements in accessing new, previously unattainable chemical matter. Performing biocatalytic processes via initial immobilization of the enzyme of interest provides significant advantages that center on enhancing stability, reusability, and productivity. Analysis of the resulting immobilized enzymes is key for optimizing biocatalytic performance, yet the current landscape for analytical technologies remains lacking. In this work, we offer a new methodology that uses unsupervised machine learning, in the form of non-negative matrix factorization (NMF), with Raman hyperspectral imaging to rapidly characterize immobilized enzymes by elucidating the spatial distributions and molecular identity of all chemical species present within the given biocatalytic system. Our proposed methodology first relies on offering a novel approach to performing NMF itself. In this manner, we offer a methodology that selects the optimal machine learning model via the determination of the most appropriate number of NMF components. Our proposed NMF methodology follows a quantitative, data-driven approach as follows—by building NMF models with varying numbers of components that range from low to high (i.e., one-ten number of components), we can select the optimal NMF model by meeting three key undisputable criteria. First, the NMF model must resolve all chemical species present. Second, of the subset of NMF models that resolve all species present, the NMF model must have the highest spectral similarity score when comparing NMF-resolved spectra with reference Raman spectra. Third, the resulting NMF-resolved chemical distributions should be in agreement with those of optical imaging. We have applied this novel NMF approach in a rigorous fashion herein, in which we evaluated diverse Raman hyperspectral imaging data sets that were collected on pantothenate kinase (PanK), an engineering enzyme evolved for optimal biocatalytic performance, immobilized to two different porous, inert resins. In all data sets analyzed, our methodology successfully resolved, both spatially and spectrally, all of the chemical species present. This resulted in up to five distinct chemical species being deconvoluted—PanK (the enzyme of interest), both acrylamide and methacrylate resins, glass substrate, adhesive glue, and Bis-Tris (used during immobilization). In total, our results demonstrate that this approach can accurately identify and spatially resolve all species within the studied enzyme immobilization process. To the best of our knowledge, this is the *first* report of a novel NMF methodology that leverages a quantitative, data-driven approach that follows a set of distinct criteria to select the optimal model based on improved component selection. Further, this is the *first* report using NMF with hyperspectral imaging for enzyme immobilization analysis. As such, our methodology offers a new powerful, data-rich experimentation tool to streamline biocatalytic process development within the pharmaceutical industry.

## Supporting Information Available

Raman spectra of all reference materials; detailed description of NMF analyses; additional discussion of NMF in comparison to other relevant machine learning methods; additional NMF analyses of PanK immobilized to acrylamide-based resin; additional NMF analyses of PanK immobilized to methacrylate-based resin; additional NMF analyses of microtomed acrylamide-based resin with immobilized PanK; cosine similarity results for chemical species not present within hyperspectral images; comprehensive cosine similarity score results (PDF)
