# Supplementary material for: Modernized Machine Learning Approach to Illuminate Enzyme Immobilization for Biocatalysis
Source: ACS Cent Sci. 2023 Sep 27;9(10):1913–26. doi: 10.1021/acscentsci.3c00757 (PMC10604017; doi:10.1021/acscentsci.3c00757)
Supplement: Supplementary file 1 — oc3c00757_si_001.pdf [file oc3c00757_si_001.pdf]

SUPPORTING INFORMATION

Modernized Machine Learning Approach to Illuminate Enzyme Immobilization for Biocatalysis

Hong Wei[1] and Joseph P. Smith[1,*]

1) Process Research & Development, MRL, Merck & Co., Inc., West Point, PA, USA 19486

**Corresponding Author: Joseph P. Smith. Phone: (484) 501-0819 ; Email: joseph.smith@merck.com ; ORCID ID: 0000-0002-0062-2534*

Supporting Information Table of Contents

Section Title	***Page(s)***
S1. Raman Spectra of Reference Materials for Enzyme Immobilization	***S2***
S2. Detailed Description of Non-Negative Matrix Factorization (NMF)	***S3-S4***
S3. Comparison of NMF With Other Relevant Machine Learning Methods	***S5-S6***
S4. NMF Analyses of PanK Immobilized onto AC Resin	***S7-S9***
S5. NMF Analyses of PanK Immobilized onto ME Resin	***S10-S14***
S6. NMF Analyses of Microtomed PanK Immobilized onto AC Resin	***S15-S17***
S7. Cosine Similarity Scores for Species Not Present in Hyperspectral Images	***S18***
S8. Comprehensive Cosine Similarity Score Summaries	***S19-S23***
S9. References for Supporting Information	***S24-27***

S1. Raman Spectra of Reference Materials for Enzyme Immobilization. Raman spectra from thirteen total reference materials relevant to the enzyme immobilization process itself (**Figure S1**) allow for us to establish an approximate reference spectral library for distinguishing the possible species present within the immobilized enzyme systems. Reference Raman spectra of both 532 nm and 785 nm excitation wavelengths are displayed.

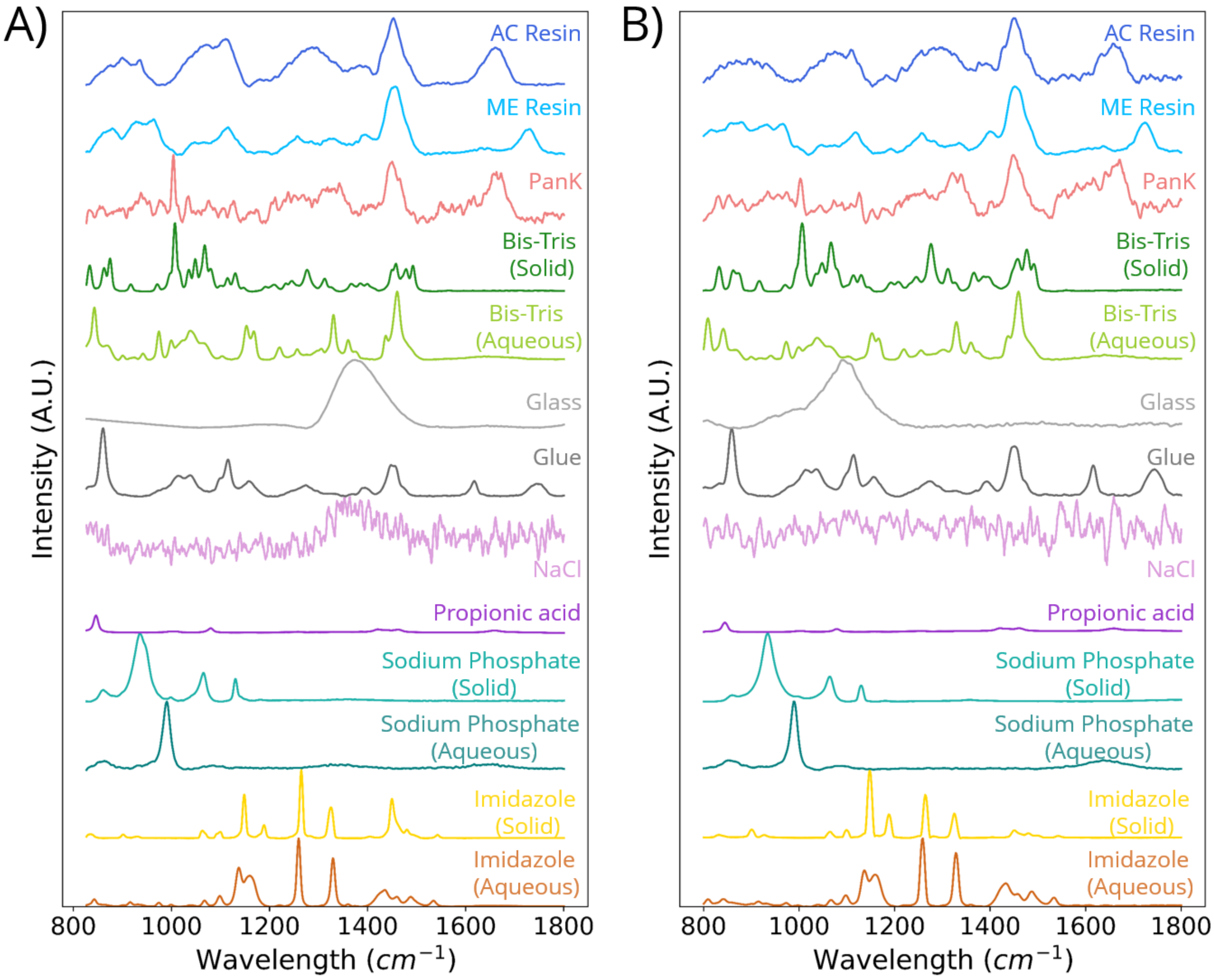

Figure S1. Representative Raman spectra obtained from reference materials of chemical species utilized within the pantothenate kinase (PanK) immobilization process using laser excitations of A) 785 nm and B) 532 nm.

S2. Detailed Description and Information Regarding Non-Negative Matrix Factorization (NMF). Non-negative matrix factorization (NMF) is an unsupervised machine learning method that has found broad application for dimension reduction, factor analysis, and feature extraction.[1-3] Detailed descriptions of NMF methodology are available throughout the literature.[4-7] NMF relies on the bilinear decomposition (***Equation S1***) of experimental data sets, as follows:

$$\boldsymbol{X} = \boldsymbol{HW'} + \boldsymbol{E} \qquad \textit{(Equation S1)}$$

where $\boldsymbol{X}$, a non-negative input matrix, decomposes into non-negative factor matrices, $\boldsymbol{H}$ and $\boldsymbol{W}$**,** along with the residual error matrix, $\boldsymbol{E}$. For decomposition, NMF requires a similarity measure, defined as $\boldsymbol{D}(\boldsymbol{X}\|\boldsymbol{HW'})$, to quantify the difference between $\boldsymbol{X}$ and its approximation $\boldsymbol{HW'}$. Quantification of this difference can be accomplished using a variety of methods, including Squared Euclidean Distance (SED), Generalized Kullback-Leibler Divergence (GKLD), or Itakura-Saito Divergence (ISD). We herein utilize an SED-based approach, as follows:

$$\boldsymbol{D}(\boldsymbol{X}\|\boldsymbol{HW'}) = \left\|\boldsymbol{X} - \boldsymbol{HW'}\right\|_{\boldsymbol{F}}^{\boldsymbol{2}} \qquad \textit{(Equation S2)}$$

where $\boldsymbol{D}$ is the difference between $\boldsymbol{X}$ and $\boldsymbol{HW'}$ and $\left\|\boldsymbol{X} - \boldsymbol{HW'}\right\|_{\boldsymbol{F}}^{\boldsymbol{2}}$ is the matrix Frobenius norm. Conveniently, this similarity measure typically may also serve as the cost function during optimization.

Prior to optimization, matrix initialization is achieved. Typically, non-negative double singular value decomposition (NNDSVD) is used for initialization (***Equation S3***), especially given that the number of chemical components is frequently less than the data (i.e., number of pixels) and features (i.e., spectral resolution and range). Specifically, a NNDSVD approach was utilized in this work. Moreover, this NNDSVD methodology also effectively promotes sparseness in the resulting NMF matrix factors, which provides superior performance for spectral feature extraction,[8] and typically can produce more statistically stable results given no randomization is contained within this approach.[9] NNDSVD is based on two singular value decomposition processes—one that approximates the data matrix and the other approximates positive sections of the resulting partial factors utilizing unit rank matrices[9], as follows:

$$\boldsymbol{A}^{(\boldsymbol{k})} := \sum_{\boldsymbol{j=1}}^{\boldsymbol{k}} \boldsymbol{\sigma_j C^{(j)}} = \boldsymbol{argmin}_{\boldsymbol{rank(G)\leq k}}\|\boldsymbol{A} - \boldsymbol{G}\| \qquad \textit{(Equation S3)}$$

where $\boldsymbol{A}^{(\boldsymbol{k})}$ is the approximation of $\boldsymbol{A}$ with respect to the Frobenius norm, $\boldsymbol{G}$ is the non-negative approximation of $\boldsymbol{A}$, and $\boldsymbol{k}$ represents the optimal rank. Every matrix $\boldsymbol{A} = \sum_{\boldsymbol{j=1}}^{\boldsymbol{r}} \boldsymbol{\sigma_j u_j v_j^{\intercal}}$, where $\boldsymbol{\sigma_1} \geq \cdots \geq \boldsymbol{\sigma_r} > \boldsymbol{0}$. $\boldsymbol{C^{(j)}} = \boldsymbol{u_j v_j^{\intercal}}$ is the unit rank matrix. NMF incorporates the non-negative constraint for both input and output data during the analysis. There are two most popular NMF optimization approaches, multiplicative optimization and coordinate descent optimization. Further, NMF approaches the optimal matrix, $\boldsymbol{W}$, that corresponds to a fixed matrix, $\boldsymbol{H}$, using a multiplicative update rule (***Equation S4 and S5***) to reduce the cost function (***Equation S2***) during optimization[10] as follows:

$$\boldsymbol{W} = \boldsymbol{W}.*(\boldsymbol{AH'})./(\boldsymbol{WHH'}) \qquad \textit{(Equation S4)}$$

$$\boldsymbol{H} = \boldsymbol{H}.*(\boldsymbol{W'A})./(\boldsymbol{W'WH}) \qquad \textit{(Equation S5)}$$

in which the operator **.*** denotes element-wise matrix multiplication, while the operator **./** denotes element-wise matrix division.

In this study, we employed the coordinate descent methodology as the optimization approach, wherein each coordinate is iteratively optimized independently according to ***Equation S6***. For NMF methodologies wholistically, the iteration goes through each i, as outlined in ***Equation S7***.

$$\boldsymbol{minimize\ \phi(x_1, x_2, \cdots, x_p)}\ \textbf{for}\ \boldsymbol{x_i \in \Omega_i} \qquad \textit{(Equation S6)}$$

$$\boldsymbol{x_i^{k+1} = argmin_{\xi} \phi(x_1^{k+1}, \cdots, x_{i-1}^{k+1}, \xi, x_{i+1}^{k}, \cdots, x_p^{k})} \qquad \textit{(Equation S7)}$$

As described in the manuscript, selecting the appropriate number of components for NMF analysis remains a challenging task. This is due to selection of too few components frequently failing to identify all species present, while selection of too many components frequently causing model overfitting. As such, we herein demonstrate within the manuscript the *first* report of a novel approach for NMF analysis that relies on a data-driven, straightforward selection of the number of NMF components. Specifically, our approach involves constructing NMF models across a range of component numbers and quantitatively comparing the resulting NMF-resolved spectra with that of reference materials to select the optimal NMF model. Using our novel approach, we utilize three distinct, quantifiable criteria to select the optimal NMF model. First, the NMF model must accurately resolve all species present. Second, the NMF model must exhibit the highest spectral similarity score between NMF-resolved spectra and that of reference materials. Third, the NMF model must demonstrate agreement and alignment between the NMF-resolved spatial distributions and the information derived from the optical image.

S3. Detailed Comparison of NMF With Other Relevant Machine Learning Methodologies. An abundance of literature has been devoted to comparing NMF with other machine learning methodologies, including multivariate curve resolution-alternating least squares (MCR-ALS).[11-20] We herein discuss some differences between NMF and MCR-ALS, including describing MCR-ALS and outlining relevant literature comparing the two machine learning methods. In short, NMF and MCR-ALS are two machine learning approaches for matrix decomposition,[12] and literature reports have been mixed in terms of performance of one approach in comparison to the other.[12, 18, 21-23] For instance, a recent literature report compared NMF and MCR-ALS with results showing NMF may generally demonstrate improved correlations relative to MCR-ALS in certain applications.[13] Similar observations between the results of NMF-based approaches and MCR-ALS have also been demonstrated in the literature.[24] Another literature report demonstrated NMF may provide improved decomposition as compared to MCR-ALS for under certain circumstances, whereas relatively similar degrees of success between methodologies were observed for other applications.[25] A high-level summary of certain differences between NMF and MCR-ALS is highlighted in **Table S1**.

Table S1. Comparison between NMF and MCR-ALS, with relevant literature included.

	**NMF**	**MCR-ALS**
Decomposition	$\boldsymbol{X = HW' + E}$	$\boldsymbol{X = HW' + E}$
Initial Estimates	NNDSVD[26]	Randomized, PCA, singular value decomposition[27]
Constraints	Non-negativity[28]	Non-negativity, spectral smoothness, rotational ambiguity constraints, equality constraints, closure constraints[29]
Optimization Strategy	Simultaneously[12]	Alternating[12]
Optimization Technique	SED, GKLD, ISD[30]	ALS[31]
Convergence Completion Rate	Potentially more straightforward, given it relies on initialization with simultaneous optimization[9]	May need more computation and more iterations to reach convergence[15]
Computational Time	Rapid (e.g., seconds)[15, 23]	Potentially more lengthy (e.g., minutes to hours)[15, 23]

To provide a straightforward comparison, we have herein provided a description of MCR-ALS. Detailed descriptions and information regarding MCR-ALS can also be found throughout the literature.[32, 33] MCR-ALS is a machine learning method commonly employed for elucidating pure response profiles of chemical components within heterogeneous samples.[34-39] MCR-ALS decomposes an experimental data matrix in a similar fashion to NMF (**Equation S1**). In the case of MCR-ALS, $\boldsymbol{E}$ is minimized in a least squares sense and the overall decomposition is accomplished using alternating least squares (ALS), which differs from the SED-based approach used in NMF. Further, MCR-ALS relies on alternating optimization, whereas NMF relies on simultaneous optimization. Thus, these two techniques allow for potentially different optimization and convergence strategies to achieve deconvolution of the desired matrix. For MCR-ALS, several techniques can be used determining the number of components, including Principal Component Analysis (PCA), prior knowledge of the data set, or other multivariate methods.[36, 38]

During optimization using ALS, various constraints are typically applied, including non-negativity, spectral smoothness, unimodality, closure constraints, or others. This is an important consideration given that MCR-ALS is affected by rotational ambiguities and thus may require additional constraints relative to NMF or other similar machine learning approaches.[40] Alternating optimization using a fast non-negative least squares approach is typically performed for MCR-ALS, in which this function solves the equation $y = xb$ using typically the constraint that b is non-negative. The inputs are x, the matrix of predictor variables, and y, the matrix of predicted variables. The output is b, corresponding to the non-negatively constrained least squares solution. This approach thus differs from that of NMF. Convergence is met through alternating application of ***Equation S8*** and ***Equation S9*** to update matrices $\boldsymbol{H}$ and $\boldsymbol{W}$, denoted as $\breve{\boldsymbol{H}}$ and $\breve{\boldsymbol{W}}$, respectively[36, 38], as follows:

$$\breve{\boldsymbol{W}} = \boldsymbol{T}'\breve{\boldsymbol{H}}/\breve{\boldsymbol{H}}'\breve{\boldsymbol{H}} \qquad \textbf{\textit{(Equation S8)}}$$

$$\breve{\boldsymbol{H}} = \boldsymbol{T}\breve{\boldsymbol{W}}/\breve{\boldsymbol{W}}'\breve{\boldsymbol{W}} \qquad \textbf{\textit{(Equation S9)}}$$

S4. NMF Analyses With Raman Hyperspectral Imaging Of PanK Immobilized Onto Acrylamide (AC) Resin. Our novel NMF methodology applied to Raman hyperspectral imaging data collected on PanK immobilized to AC resin under 532 nm excitation is able to successfully resolve the spatial distributions and molecular identify of both resin and enzyme (**Figure S2**). For this data set, 3660 total Raman spectra were collected in a 57 × 55 spectral grid. Using the novel NMF analysis outlined in the manuscript, chemical images and corresponding Raman spectra for each individual species present within the hyperspectral image were deconvoluted. NMF-resolved spectra were quantitatively compared to reference spectra using a cosine similarity score to identify each resolved species.

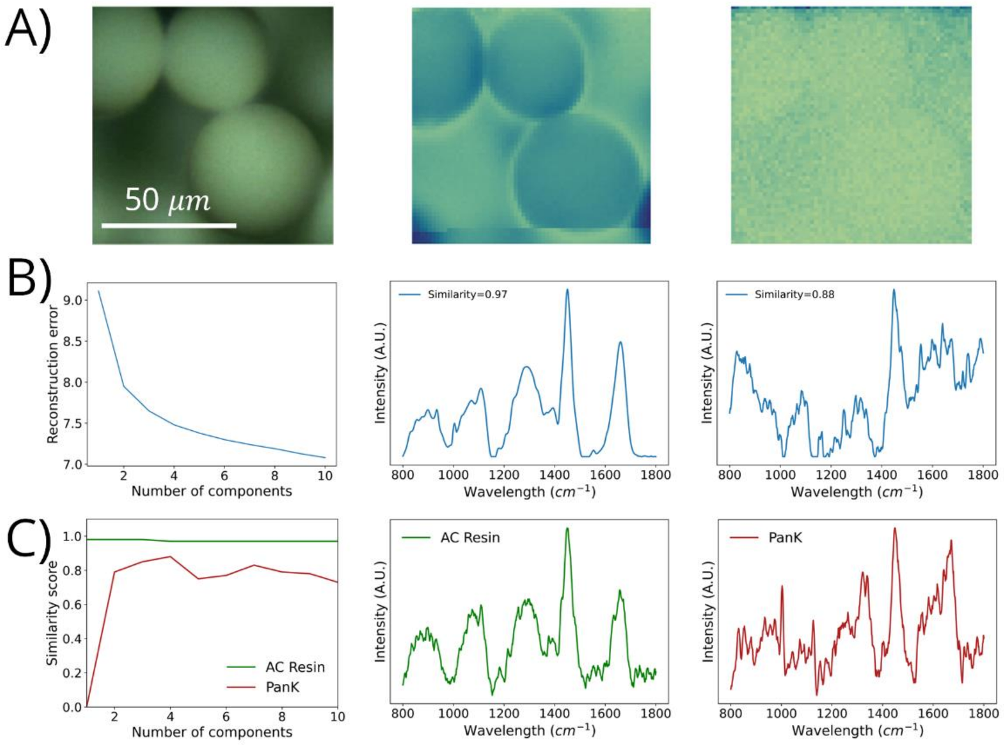

Figure S2. Summary of results for NMF with Raman hyperspectral imaging of PanK immobilized to acrylamide-based (AC) resin using 532 nm excitation. A) Optical image and NMF-resolved chemical images of AC resin and PanK (left to right). B) Relative reconstruction error (RRE) plot and NMF-resolved Raman spectra that correspond to AC resin and PanK (left to right). For the RRE plot, the x-axis represents the number of components used to construct independent NMF models and the y-axis represents the relative reconstruction error. For the NMF-resolved Raman spectra, the cosine spectral similarity score is displayed within each spectra. C) Spectral similarity plot with increasing number of NMF components and Raman spectra of reference materials—AC resin and PanK (left to right).

In order to select the optimal NMF model using our new methodology, ten independent NMF models were constructed with the number of components ranging from one to ten.

Comprehensive results for these ten independent NMF models are displayed with respect to both the resolved concentration profiles (**Figure S3**) and corresponding resolved spectra (**Figure S4**).

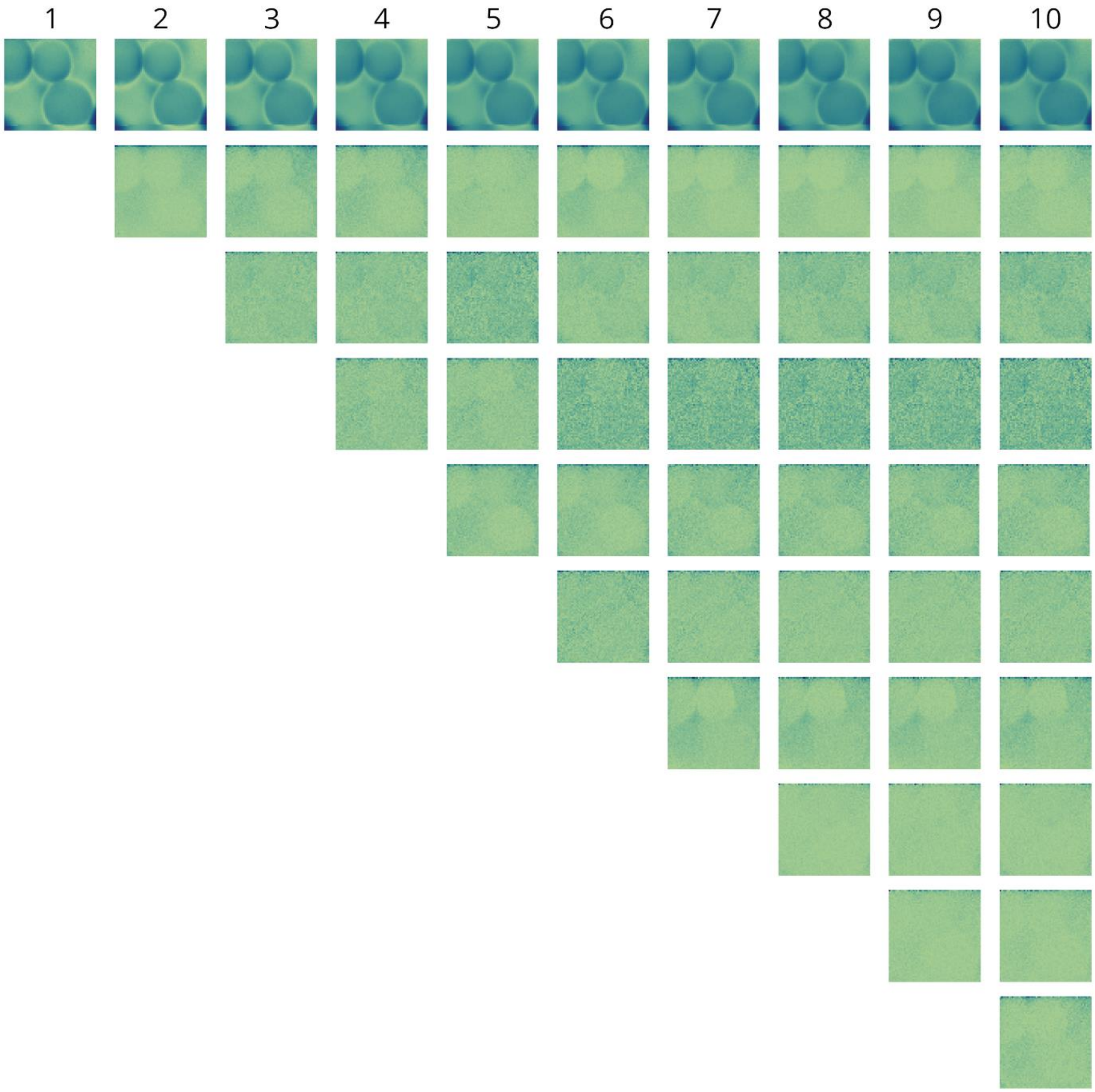

Figure S3. NMF-resolved concentration profiles formed into chemical images for PanK immobilized onto acrylamide-based (AC) resin under 532 nm excitation. NMF models were constructed with varying numbers of components ranging from one to ten, in which the number at the top of each column represents the number of components used to construct the independent NMF model. Each column displays the chemical images resulting from the NMF analysis using the respective number of components.

Using a one component NMF model, the resin is resolved. This is evidenced by the high spectral similar score of 0.97 between the NMF-resolved spectrum and the reference Raman spectrum, and the NMF-resolved chemical distribution demonstrating agreement with the resin

locations with respect to the optical image. Further increasing the number of components to two yields the resolution of PanK, in which a spectral similarity score of 0.79 is observed when comparing the NMF-resolved spectrum with the PanK reference spectrum. No additional chemical species were resolved using NMF models constructed with more than two components. However, the similarity score for PanK increases from 0.79 to 0.88 when using a four component NMF model as compared with a two component NMF model. As such, the optimal NMF model for this data set is determined to be a four component model.

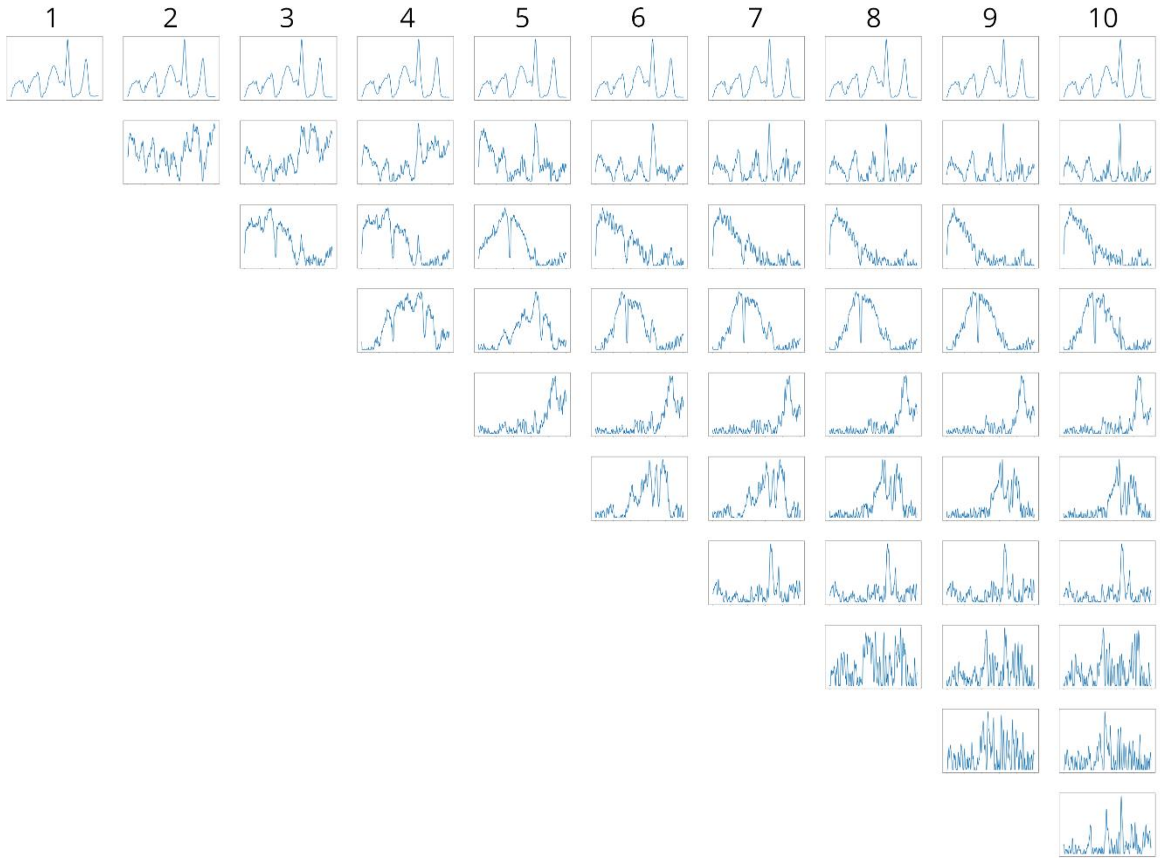

Figure S4. NMF-resolved Raman spectra for PanK immobilized onto acrylamide-based (AC) resin under 532 nm excitation. NMF models were constructed with varying numbers of components ranging from one to ten, in which the number at the top of each column represents the number of components used to construct the independent NMF model. Each column displays the NMF-resolved Raman spectra resulting from the NMF analysis using the respective number of components. The x axis for each individual spectrum is wavelength (cm^{-1}), ranging from 800 cm^{-1} to 1800 cm^{-1}. The y axis for each individual spectrum represents intensity (arbitrary units).

S5. NMF Analyses With Raman Hyperspectral Imaging Of PanK Immobilized Onto Methacrylate (ME) Resin. The novel NMF methodology proposed in this work applied to Raman hyperspectral imaging data collected on PanK immobilized to ME resin under 785 nm excitation is able to successfully resolve the spatial distributions and molecular identity of resin, enzyme, glass substrate, and Bis-Tris. Comprehensive results for the ten independent NMF models used to determine the optimal number of components are displayed in terms of both the NMF-resolved chemical images (**Figure S5**) and corresponding NMF-resolved Raman spectra (**Figure S6**).

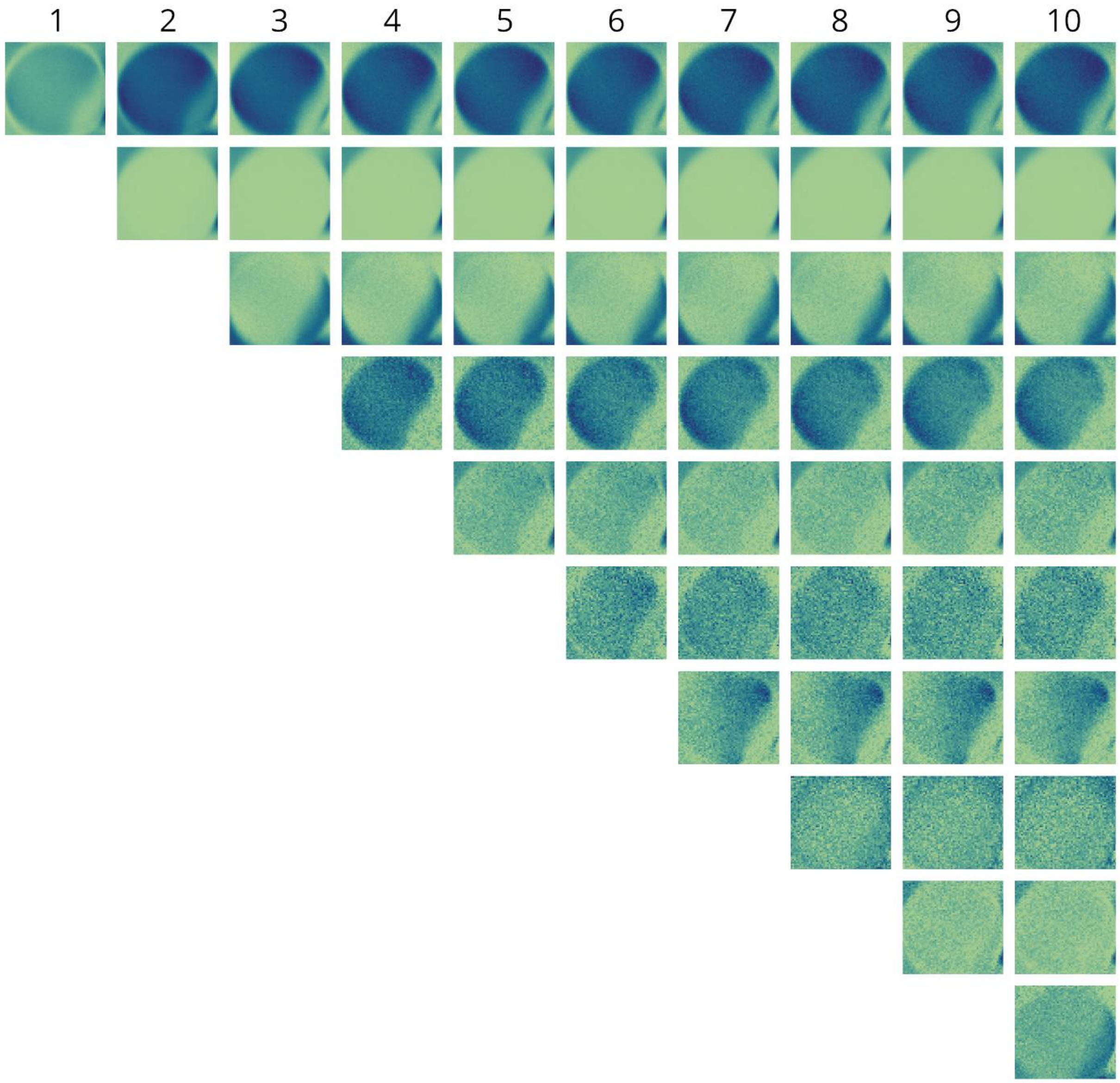

Figure S5. NMF-resolved concentration profiles formed into chemical images for PanK immobilized onto methacrylate-based (ME) resin under 785 nm excitation. NMF models were constructed with varying numbers of components ranging from one to ten, in which the number at the top of each column represents the number of components used to construct the

independent NMF model. Each column displays the chemical images resulting from the NMF analysis using the respective number of components.

Using a three component NMF model, resin and glass were resolved with spectral similarities of 0.97 and 1.00, respectively. Resolution of Bis-Tris and PanK was accomplished by increasing the number of components to four, resulting in a spectral similarity of 0.81 observed for Bis-Tris and 0.74 observed for PanK. Increasing the number of components beyond four did not result in resolution of any additional species. Given our novel approach to NMF analysis as described in the manuscript, the optimal NMF model was selected to be four components.

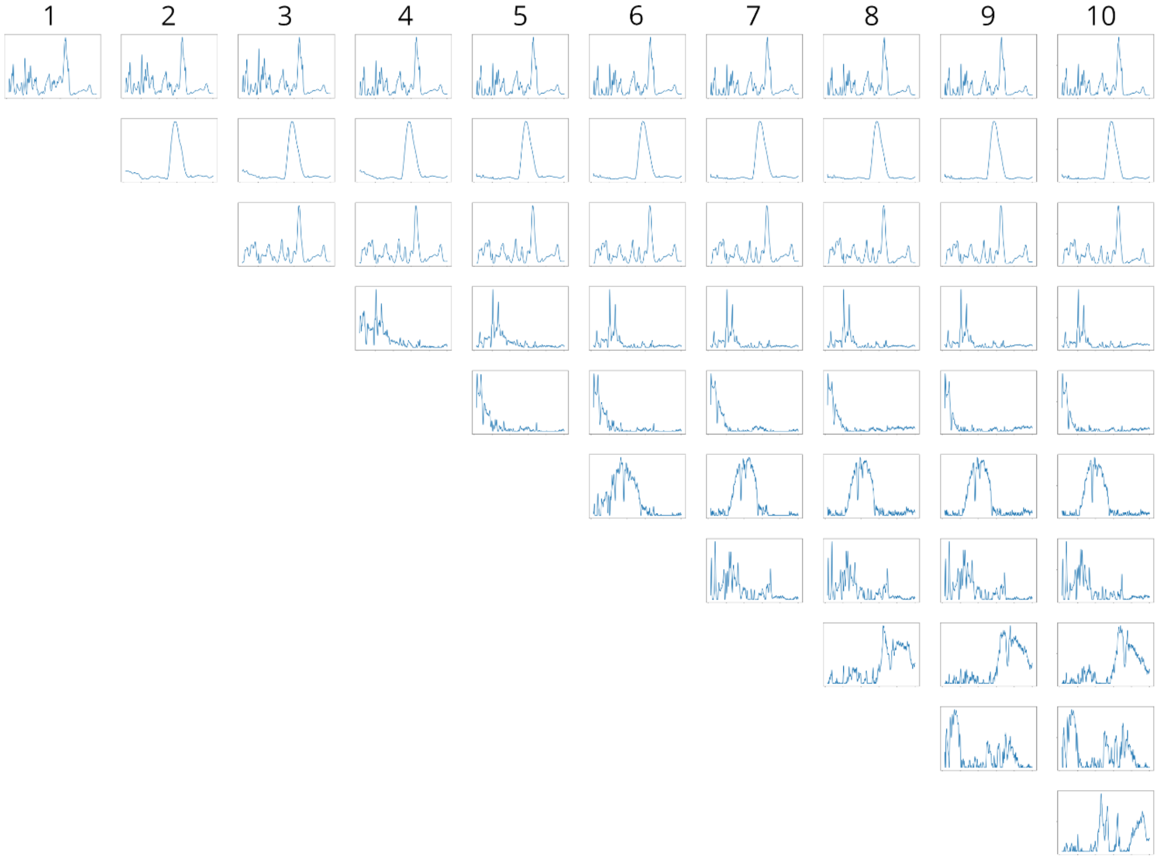

Figure S6. NMF-resolved Raman spectra for PanK immobilized onto methacrylate-based (ME) resin under 785 nm excitation. NMF models were constructed with varying numbers of components ranging from one to ten, in which the number at the top of each column represents the number of components used to construct the independent NMF model. Each column displays the NMF-resolved Raman spectra resulting from the NMF analysis using the respective number of components. The x axis for each individual spectrum is wavelength (cm^{-1}), ranging from 800 cm^{-1} to 1800 cm^{-1}. The y axis for each individual spectrum represents intensity (arbitrary units).

Our NMF methodology applied to Raman hyperspectral imaging data collected on PanK immobilized to ME resin under 532 nm excitation was able to successfully resolve the spatial

distributions and molecular identity of resin, enzyme, glass substrate, and Bis-Tris. Comprehensive results for the ten independent NMF models used to determine the optimal number of components are displayed as the NMF-resolved chemical images (**Figure S7**) and corresponding NMF-resolved Raman spectra (**Figure S8**).

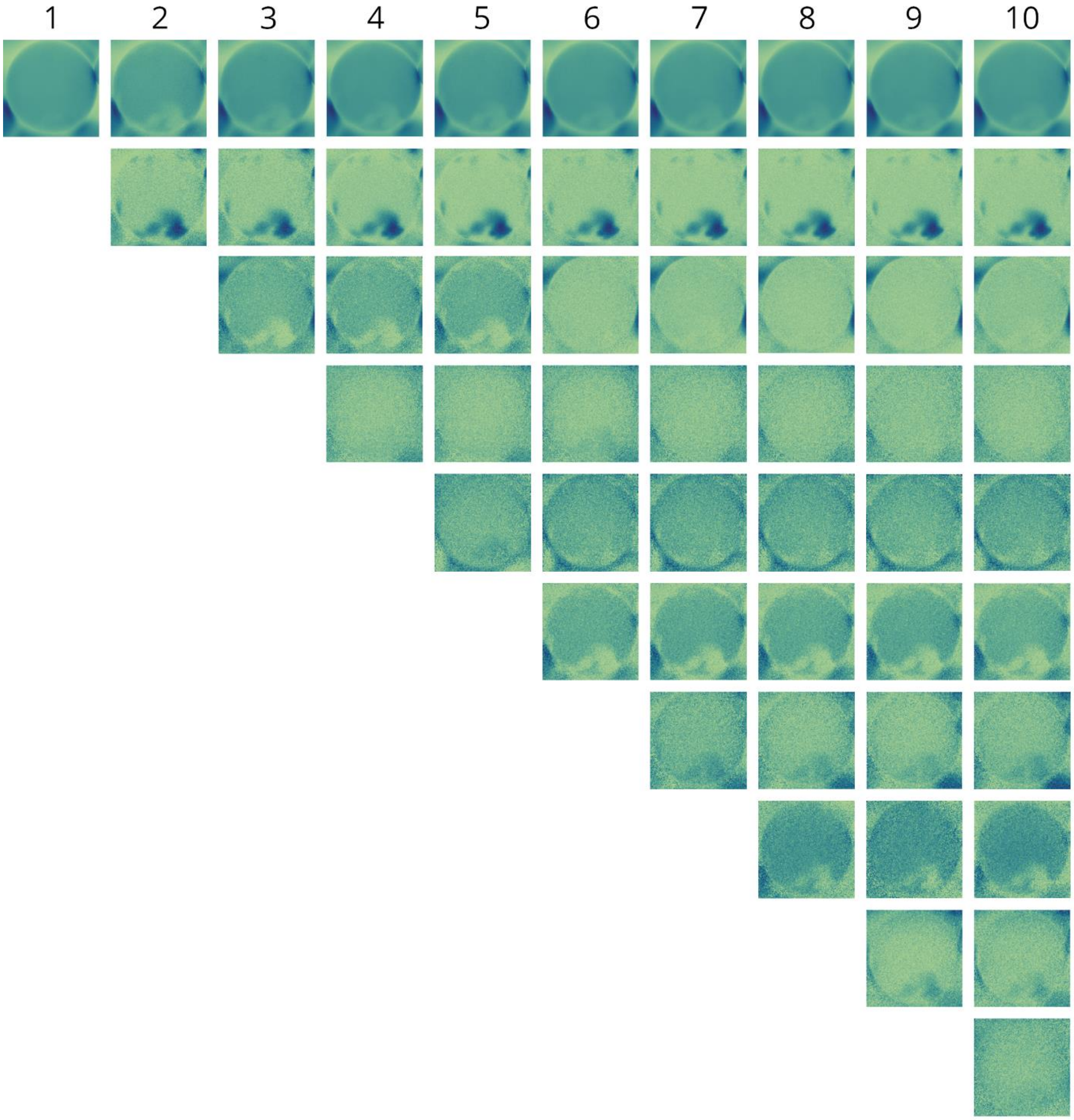

Figure S7. NMF-resolved concentration profiles formed into chemical images for PanK immobilized onto methacrylate-based (ME) resin under 532 nm excitation. NMF models were constructed with varying numbers of components ranging from one to ten, in which the number at the top of each column represents the number of components used to construct the

independent NMF model. Each column displays the chemical images resulting from the NMF analysis using the respective number of components.

Using a one component NMF model, resin was resolved and resulted in a 0.99 spectral similarity score. Using a two component NMF model, resin and Bis-Tris were resolved with spectral similarity scores of 0.99 and 0.66, respectively. Using a six component NMF model, resin, Bis-Tris, and glass were resolved with spectral similarities of 0.99, 0.80, and 0.93, respectively. This increase from two to six number of components resulted in a significant improvement in Bis-Tris resolution, as evidenced by the spectral similarity scores reported. Using an eight component NMF model, resin, Bis-Tris, glass, and PanK were resolved with spectral similarity scores of 0.98, 0.81, 0.91, 0.80, respectively. No additional species were resolved when the numbers of components were increased above eight. As such, the optimal NMF model selected was using an eight component model, in which all species were resolved (resin, Bis-Tris, glass, and PanK), high spectral similarities were observed, and the NMF-resolved chemical images were in great agreement with the optical image.

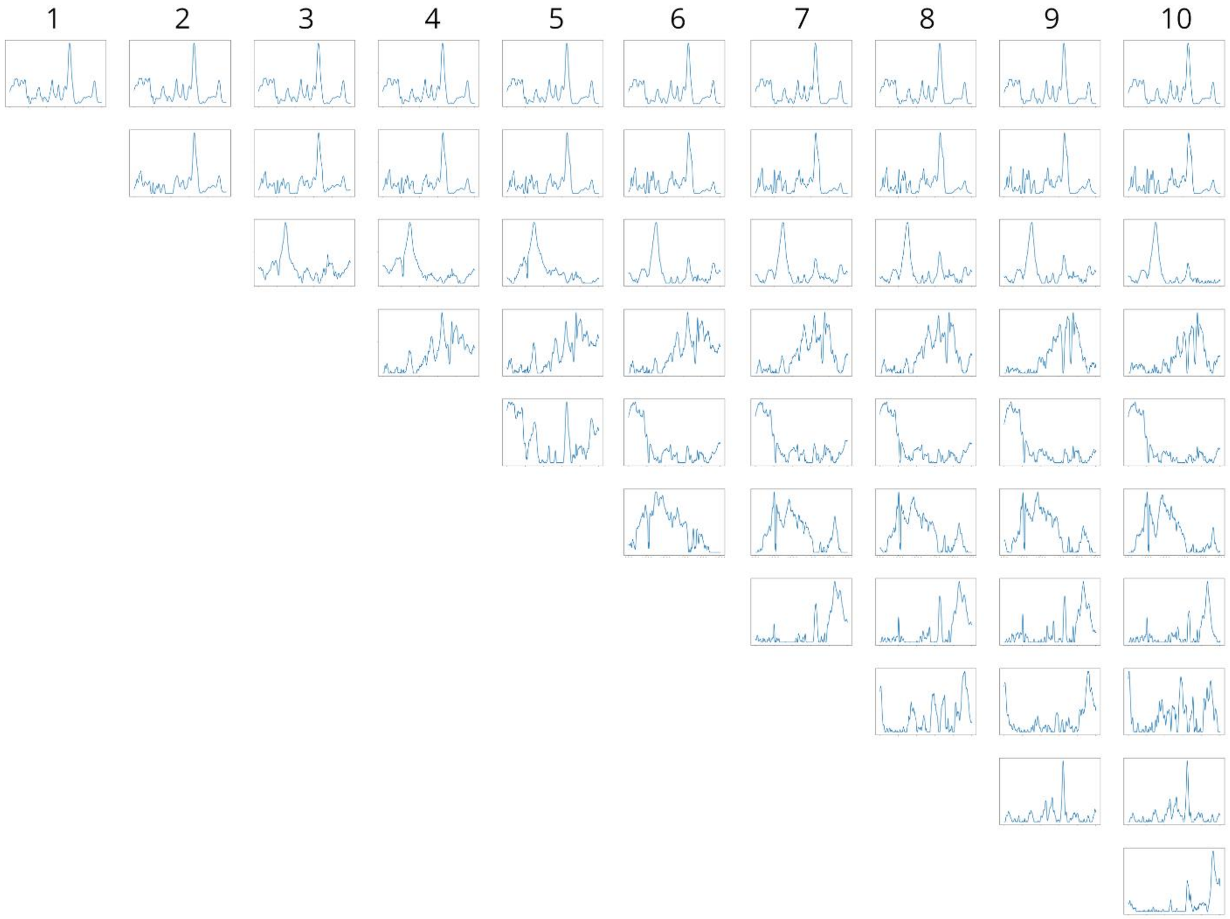

Figure S8. NMF-resolved Raman spectra for PanK immobilized onto methacrylate-based (ME) resin under 532 nm excitation. NMF models were constructed with varying numbers of components ranging from one to ten, in which the number at the top of each column represents the number of components used to construct the independent NMF model. Each column

displays the NMF-resolved Raman spectra resulting from the NMF analysis using the respective number of components. The x axis for each individual spectrum is wavelength (cm^{-1}), ranging from 800 cm^{-1} to 1800 cm^{-1}. The y axis for each individual spectrum represents intensity (arbitrary units).

S6. NMF Analyses With Raman Hyperspectral Imaging Of Microtomed PanK Immobilized Onto Acrylamide (AC) Resin. To investigate if PanK is present both within the resin beads or is primarily located on the surface, the immobilized enzyme systems were microtomed prior to Raman hyperspectral imaging. This microtome sample preparation allows for a representative cross section of PanK immobilized to the AC resin. The novel NMF methodology presented herein applied to the Raman hyperspectral imaging data collected on microtomed PanK immobilized to AC resin under 785 nm excitation was able to successfully resolve the spatial distributions and molecular identity of resin, enzyme, glass substrate, and Bis-Tris. Comprehensive results for the ten independent NMF models used to determine the optimal number of components are displayed as the NMF-resolved chemical images (**Figure S9**) and corresponding NMF-resolved Raman spectra (**Figure S10**).

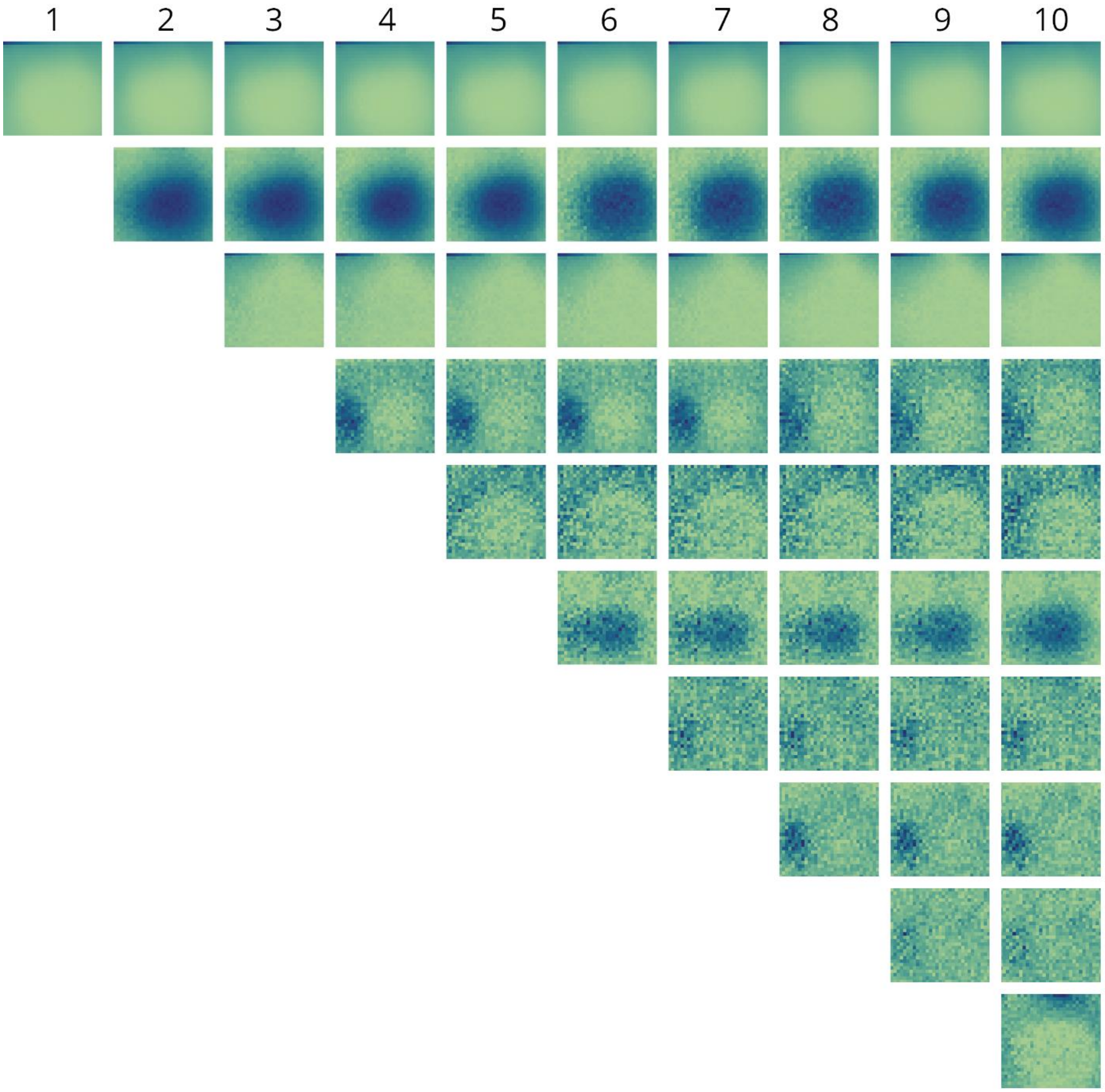

Figure S9. NMF-resolved concentration profiles formed into chemical images for microtomed PanK immobilized onto acrylamide-based (AC) resin under 785 nm excitation. NMF models were constructed with varying numbers of components ranging from one to ten, in which the number at the top of each column represents the number of components used to construct the independent NMF model. Each column displays the chemical images resulting from the NMF analysis using the respective number of components.

Using a one component NMF model, glue was resolved and resulted in a 1.00 spectral similarity score. Glue is used as an adhesive for microtome analysis, so resolution of this species is intuitive and consistent with sample preparation procedures performed herein. Using a two component NMF model, glue and resin were resolved with spectral similarity scores of 1.00 and 0.96, respectively. Using a four component NMF model, glue, resin, and Bis-Tris were resolved with spectral similarity scores of 1.00, 0.96, and 0.69, respectively. Using a six component NMF model, glue, resin, Bis-Tris, and PanK were resolved with spectral similarities of 1.00, 0.94, 0.69, and 0.78, respectively. No additional species were resolved when the numbers of components were further increased. As such, the optimal NMF model selected was using a six component model, in which all species were resolved (resin, PanK, Bis-Tris, and glue), high spectral similarities were observed, and the NMF-resolved chemical images were in great agreement with the optical image.

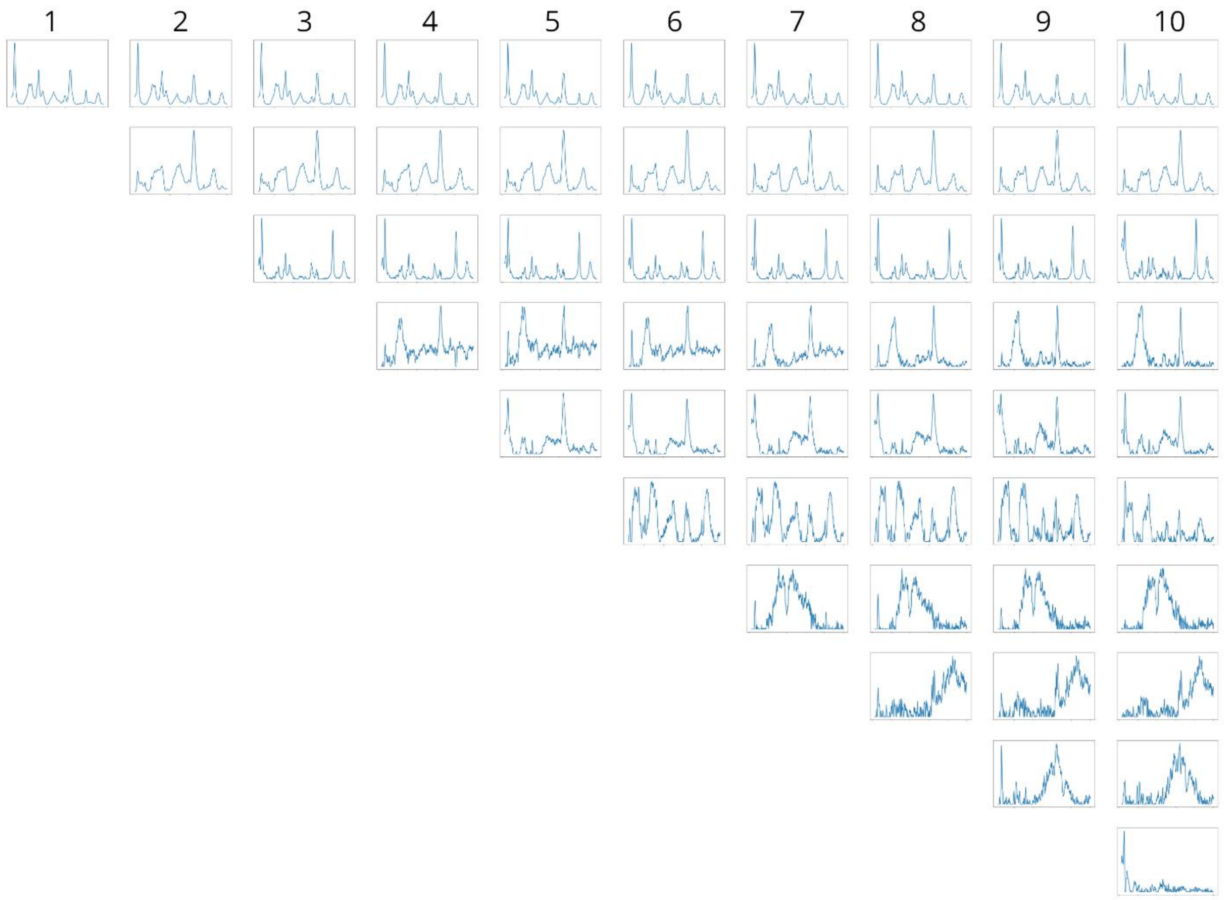

***Figure S10.** NMF-resolved Raman spectra for microtomed PanK immobilized onto acrylamide-based (AC) resin under 785 nm excitation. NMF models were constructed with varying numbers of components ranging from one to ten, in which the number at the top of each column represents the number of components used to construct the independent NMF model. Each column displays the NMF-resolved Raman spectra resulting from the NMF analysis using the respective number of components. The x axis for each individual spectrum is wavelength (cm^{-1}), ranging from 800 cm^{-1} to 1800 cm^{-1}. The y axis for each individual spectrum represents intensity (arbitrary units).*

S7. Cosine Similarity Score Results for Species Not Present within Hyperspectral Images. The similarity scores between NMF-resolved spectra and reference spectra of species not present within the data (i.e., sodium phosphate, imidazole and propionic acid) were determined as a control. Results show that all spectral similarity scores are <0.5. This observation allows for an approximate, estimated threshold to enable improved species identification and interpretation of NMF models. In addition, the behavior of the spectral similarity score trends (**Figure S11**) for species not present as compared to resolved species (i.e., AC resin, glass, Bis-Tris, PanK) is informative. For the resolved species, the spectral similarities are both high in number and are statistically stable, as evidenced by the relatively flat and constant trends in **Figure S11**. For the species not present generated as a control (i.e., sodium phosphate, imidazole and propionic acid), the spectral similarities are both low in number and are not statistically stable, as evidenced by fluctuating and changing trends in **Figure S11**. We also note that for species that are resolved, we see a sharp increase in the spectral similarity score upon initial NMF resolution, as expected. This represents the first time an NMF-resolved spectrum is similar to that a reference. For the species not present, however, we do not observe this sharp increase upon initial resolution, and subsequent to that, we observe high variance in the spectral similarity score. This is intuitive given that these observations are also consistent with noise or other background spectral features, helping further provide insights into evaluating the similarities of NMF-resolved spectra and reference spectra.

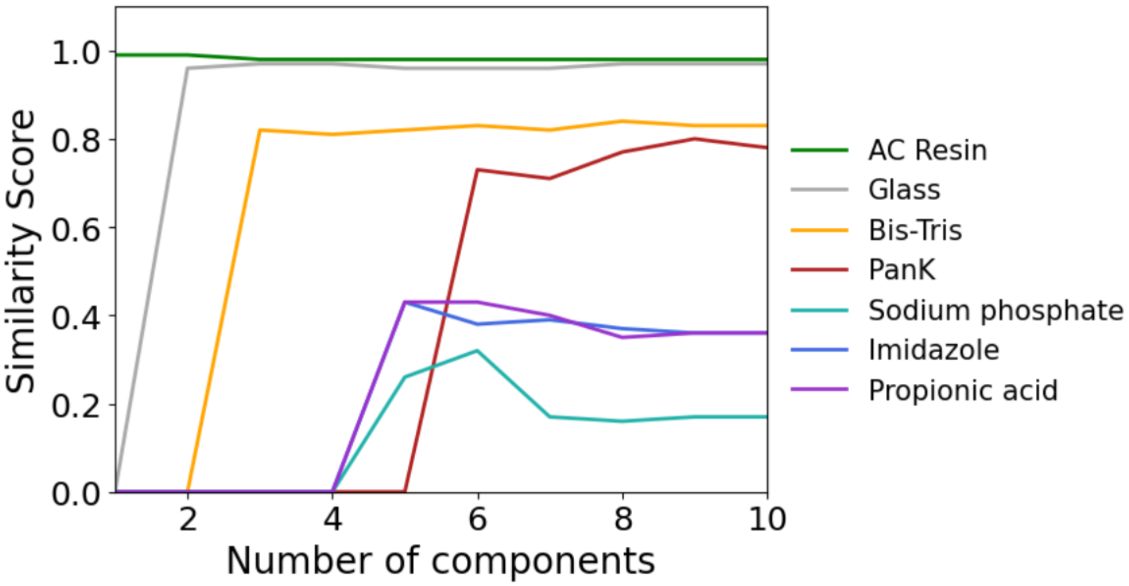

Figure S11. The similarity score summarization for the chemical species resolved by NMF model (AC resin, glass, Bis-Tris, and PanK) and chemical species involved in the enzyme immobilization process but not in the final product (sodium phosphate, imidazole and propionic acid).

S8. Comprehensive Cosine Similarity Scores Summaries. Cosine spectral similarity scores were thus determined for each NMF-resolved spectra across all potential reference Raman spectra for all independent NMF models. Results for cosine similarity score determinations are displaying in full for PanK immobilized to AC resin under 785 nm excitation (**Table 1**). Additionally, for the four other data sets analyzed herein, full results of the cosine similarity score determinations are displayed within the Supporting Information—spectral similarity from NMF-resolved spectra compared to that of reference material for PanK immobilized to ME resin under 785 nm excitation (**Table S1**), spectral similarity from NMF-resolved spectra compared to that of reference material for PanK immobilized to ME resin under 532 nm excitation (**Table S2**), spectral similarity from NMF-resolved spectra compared to that of reference material for PanK immobilized to AC resin under 532 nm excitation (**Table S3**), and spectral similarity from NMF-resolved spectra compared to that of reference material for microtomed PanK immobilized to AC resin under 785 nm excitation (**Table S4**).

Table S2. Spectral similarity of NMF-resolved spectra and reference spectra for hyperspectral imaging (785 nm excitation) of PanK immobilized to methacrylate resin.

**NMF Model Number**	**Component Number**	**Spectral Similarity**			
		Methacrylate Resin	*PanK*	*Bis-Tris*	*Glass*
NMF model 1	1	0.88	0.77	0.81	0.54
NMF model 2	1	0.86	0.77	0.84	0.38
	2	0.57	0.54	0.29	0.99
NMF model 3	1	0.83	0.75	0.87	0.37
	2	0.54	0.52	0.29	1.00
	3	0.97	0.78	0.56	0.42
**NMF model 4***	1	0.83	***0.74***	0.81	0.38
	2	0.53	0.52	0.28	***1.00***
	3	***0.97***	0.78	0.53	0.42
	4	0.49	0.49	***0.81***	0.18
NMF model 5	1	0.83	0.73	0.80	0.38
	2	0.51	0.50	0.26	0.99
	3	0.97	0.78	0.53	0.42
	4	0.39	0.50	0.82	0.14
	5	0.44	0.29	0.41	0.19
NMF model 6	1	0.83	0.73	0.80	0.38
	2	0.50	0.50	0.26	0.99
	3	0.97	0.77	0.52	0.42
	4	0.35	0.45	0.82	0.12
	5	0.43	0.28	0.40	0.17
	6	0.45	0.55	0.51	0.21
NMF model 7	1	0.83	0.72	0.78	0.37
	2	0.50	0.50	0.25	0.99
	3	0.97	0.77	0.52	0.42
	4	0.34	0.44	0.82	0.13
	5	0.42	0.28	0.35	0.21
	6	0.38	0.53	0.39	0.22
	7	0.54	0.5	0.73	0.22
NMF model 8	1	0.83	0.71	0.79	0.37
	2	0.49	0.48	0.25	0.99
	3	0.97	0.75	0.52	0.42
	4	0.34	0.44	0.82	0.12
	5	0.42	0.31	0.36	0.18
	6	0.37	0.53	0.38	0.21
	7	0.54	0.50	0.74	0.23
	8	0.62	0.71	0.36	0.30
NMF model 9	1	0.83	0.71	0.79	0.37
	2	0.49	0.47	0.25	0.99
	3	0.97	0.74	0.52	0.42
	4	0.34	0.46	0.84	0.14
	5	0.36	0.27	0.38	0.18
	6	0.36	0.52	0.38	0.20
	7	0.53	0.49	0.74	0.22
	8	0.54	0.66	0.33	0.27
	9	0.56	0.50	0.22	0.28
NMF model 10	1	0.83	0.69	0.80	0.37
	2	0.48	0.47	0.25	0.99
	3	0.97	0.71	0.53	0.41
	4	0.33	0.48	0.82	0.13
	5	0.36	0.24	0.37	0.17
	6	0.36	0.50	0.39	0.17
	7	0.47	0.48	0.75	0.19
	8	0.59	0.66	0.4	0.31
	9	0.57	0.54	0.24	0.36
	10	0.52	0.70	0.27	0.25

*Note: The optimal NMF model is denoted by *. The spectral similarity score for each final resolved species is bolded and italicized for ease of visualization.*

Table S3. Results of spectral similarity from NMF-resolved spectra and reference spectra using hyperspectral imaging (532 nm excitation) of PanK immobilized to methacrylate resin.

NMF Model Number	Component Number	Spectral Similarity			
		Methacrylate Resin	*PanK*	*Bis-Tris*	*Glass*
NMF model 1	1	0.99	0.77	0.58	0.33
NMF model 2	1	0.99	0.76	0.57	0.34
	2	0.94	0.76	0.66	0.25
NMF model 3	1	0.99	0.75	0.56	0.30
	2	0.93	0.78	0.71	0.32
	3	0.49	0.63	0.49	0.89
NMF model 4	1	0.99	0.75	0.56	0.29
	2	0.92	0.72	0.75	0.34
	3	0.49	0.50	0.52	0.94
	4	0.58	0.86	0.35	0.31
NMF model 5	1	0.99	0.75	0.56	0.28
	2	0.89	0.71	0.76	0.34
	3	0.40	0.48	0.55	0.95
	4	0.63	0.88	0.41	0.27
	5	0.86	0.65	0.46	0.47
NMF model 6	1	0.99	0.74	0.55	0.29
	2	0.88	0.70	0.80	0.35
	3	0.57	0.49	0.52	0.93
	4	0.61	0.88	0.39	0.21
	5	0.69	0.52	0.38	0.27
	6	0.52	0.64	0.56	0.74
NMF model 7	1	0.99	0.73	0.55	0.29
	2	0.86	0.70	0.81	0.34
	3	0.56	0.50	0.52	0.93
	4	0.56	0.81	0.43	0.22
	5	0.70	0.55	0.39	0.31
	6	0.42	0.66	0.60	0.65
	7	0.47	0.68	0.21	0.12
**NMF model 8***	1	***0.98***	0.73	0.55	0.29
	2	0.86	0.70	***0.81***	0.33
	3	0.59	0.55	0.53	***0.91***
	4	0.56	0.80	0.44	0.20
	5	0.68	0.52	0.38	0.29
	6	0.43	0.63	0.61	0.67
	7	0.51	***0.74***	0.27	0.14
	8	0.51	0.48	0.31	0.27
NMF model 9	1	0.99	0.73	0.55	0.29
	2	0.85	0.69	0.82	0.33
	3	0.58	0.53	0.53	0.92
	4	0.44	0.69	0.41	0.15
	5	0.67	0.50	0.40	0.32
	6	0.42	0.61	0.61	0.67
	7	0.51	0.76	0.29	0.16
	8	0.47	0.47	0.18	0.18
	9	0.68	0.67	0.43	0.23
NMF model 10	1	0.99	0.73	0.55	0.29
	2	0.85	0.68	0.82	0.32
	3	0.45	0.40	0.49	0.96
	4	0.47	0.71	0.38	0.18
	5	0.64	0.48	0.35	0.29
	6	0.44	0.56	0.60	0.59
	7	0.40	0.76	0.28	0.15
	8	0.48	0.58	0.35	0.28
	9	0.66	0.68	0.44	0.24
	10	0.54	0.45	0.21	0.12

*Note: The optimal NMF model is denoted by *. The spectral similarity score for each final resolved species is bolded and italicized for ease of visualization.*

Table S4. Results of spectral similarity from NMF-resolved spectra and reference spectra using hyperspectral imaging (532 nm excitation) of PanK immobilized to acrylamide resin.

NMF Model Number	Component Number		
		Acrylamide Resin	PanK
NMF model 1	1	0.98	0.89
NMF model 2	1	0.98	0.88
	2	0.71	0.79
NMF model 3	1	0.98	0.88
	2	0.74	0.85
	3	0.74	0.66
**NMF model 4***	1	***0.97***	0.87
	2	0.80	***0.88***
	3	0.72	0.63
	4	0.80	0.77
NMF model 5	1	0.97	0.87
	2	0.79	0.75
	3	0.69	0.60
	4	0.79	0.77
	5	0.56	0.72
NMF model 6	1	0.97	0.87
	2	0.84	0.77
	3	0.67	0.63
	4	0.73	0.62
	5	0.56	0.73
	6	0.59	0.68
NMF model 7	1	0.97	0.87
	2	0.88	0.83
	3	0.59	0.56
	4	0.73	0.61
	5	0.57	0.72
	6	0.59	0.69
	7	0.67	0.67
NMF model 8	1	0.97	0.87
	2	0.88	0.79
	3	0.57	0.53
	4	0.70	0.59
	5	0.55	0.71
	6	0.61	0.68
	7	0.68	0.67
	8	0.70	0.78
NMF model 9	1	0.97	0.87
	2	0.87	0.78
	3	0.55	0.52
	4	0.67	0.56
	5	0.56	0.71
	6	0.62	0.68
	7	0.71	0.68
	8	0.71	0.76
	9	0.68	0.73
NMF model 10	1	0.97	0.87
	2	0.83	0.73
	3	0.61	0.56
	4	0.69	0.58
	5	0.58	0.71
	6	0.61	0.68
	7	0.65	0.63
	8	0.71	0.79
	9	0.71	0.70
	10	0.67	0.65

*Note: The optimal NMF model is denoted by *. The spectral similarity score for each final resolved species is bolded and italicized for ease of visualization.*

Table S5. Spectral similarity of NMF-resolved spectra and reference spectra using hyperspectral imaging (785 nm excitation) of microtomed PanK and acrylamide resin.

NMF Model Number	Component Number	Spectral Similarity			
		Acrylamide Resin	*PanK*	*Bis-Tris*	*Glue*
NMF model 1	1	0.69	0.61	0.66	1.00
NMF model 2	1	0.63	0.56	0.64	1.00
	2	0.96	0.87	0.66	0.70
NMF model 3	1	0.64	0.56	0.65	1.00
	2	0.96	0.87	0.65	0.69
	3	0.39	0.39	0.37	0.77
NMF model 4	1	0.64	0.56	0.64	1.00
	2	0.96	0.86	0.65	0.70
	3	0.38	0.39	0.39	0.79
	4	0.80	0.86	0.69	0.66
NMF model 5	1	0.64	0.56	0.64	1.00
	2	0.96	0.86	0.65	0.71
	3	0.36	0.37	0.37	0.77
	4	0.77	0.83	0.70	0.71
	5	0.73	0.70	0.61	0.79
**NMF model 6***	1	0.64	0.55	0.64	***1.00***
	2	***0.94***	0.84	0.65	0.74
	3	0.39	0.39	0.38	0.79
	4	0.77	0.83	***0.69***	0.74
	5	0.68	0.65	0.60	0.81
	6	0.88	***0.78***	0.55	0.48
NMF model 7	1	0.63	0.55	0.64	1.00
	2	0.94	0.83	0.65	0.75
	3	0.39	0.39	0.37	0.76
	4	0.72	0.79	0.64	0.68
	5	0.69	0.65	0.56	0.74
	6	0.88	0.78	0.56	0.48
	7	0.71	0.64	0.49	0.54
NMF model 8	1	0.63	0.55	0.64	1.00
	2	0.93	0.82	0.66	0.75
	3	0.39	0.39	0.37	0.77
	4	0.67	0.64	0.73	0.68
	5	0.71	0.67	0.57	0.75
	6	0.85	0.75	0.47	0.41
	7	0.65	0.61	0.44	0.54
	8	0.51	0.68	0.28	0.40
NMF model 9	1	0.63	0.54	0.63	1.00
	2	0.93	0.82	0.65	0.73
	3	0.41	0.42	0.40	0.77
	4	0.63	0.60	0.73	0.64
	5	0.67	0.64	0.57	0.74
	6	0.78	0.68	0.45	0.44
	7	0.65	0.59	0.42	0.49
	8	0.56	0.71	0.33	0.44
	9	0.65	0.66	0.48	0.56
NMF model 10	1	0.63	0.54	0.63	0.99
	2	0.93	0.83	0.64	0.70
	3	0.36	0.40	0.39	0.74
	4	0.59	0.56	0.71	0.60
	5	0.74	0.69	0.64	0.78
	6	0.74	0.60	0.46	0.56
	7	0.66	0.62	0.42	0.44
	8	0.56	0.71	0.29	0.35
	9	0.60	0.65	0.41	0.45
	10	0.33	0.35	0.36	0.52

*Note: The optimal NMF model is denoted by *. The spectral similarity score for each final resolved species is bolded and italicized for ease of visualization.*

S9. References for Supporting Information

1. You, M.; Wang, H.; Liu, Z.; Chen, C.; Liu, J.; Xu, X.-H.; Qiu, Z.-M., Novel feature extraction method for cough detection using NMF. *IET Signal Processing* **2017,** *11* (5), 515-520.

2. Ludeña-Choez, J.; Choquehuanca-Zevallos, J. J.; Mayhua-López, E., Sensor nodes fault detection for agricultural wireless sensor networks based on NMF. *Computers and Electronics in Agriculture* **2019,** *161*, 214-224.

3. Zhou, J.; Zhang, S.; Mei, H.; Wang, D., A method of facial expression recognition based on Gabor and NMF. *Pattern Recognition and Image Analysis* **2016,** *26* (1), 119-124.

4. Yan, H.; Fan, W.; Chen, X.; Wang, H.; Qin, C.; Jiang, X., Component spectra extraction and quantitative analysis for preservative mixtures by combining terahertz spectroscopy and machine learning. *Spectrochimica Acta Part A: Molecular and Biomolecular Spectroscopy* **2022,** *271*, 120908.

5. Szymańska-Chargot, M.; Pieczywek, P. M.; Chylińska, M.; Zdunek, A., Hyperspectral image analysis of Raman maps of plant cell walls for blind spectra characterization by nonnegative matrix factorization algorithm. *Chemometrics and Intelligent Laboratory Systems* **2016,** *151*, 136-145.

6. Anbumalar, S.; Anandanatarajan, R.; Rameshbabu, P., Sparse Non-negative Matrix Factorization and its Application in Overlapped Chromatograms Separation. *International Journal of Computer Applications* **2013,** *63,* 1-10.

7. Lee, D. D.; Seung, H. S., Learning the parts of objects by non-negative matrix factorization. *Nature* **1999,** *401* (6755), 788-791.

8. Zushi, Y., NMF-Based Spectral Deconvolution with a Web Platform GC Mixture Touch. *ACS Omega* **2021,** *6* (4), 2742-2748.

9. Boutsidis, C.; Gallopoulos, E., SVD based initialization: A head start for nonnegative matrix factorization. *Pattern Recognition* **2008,** *41* (4), 1350-1362.

10. Huang, S.; Zhao, Y.; Hu, C.; Qin, B. In *Nonnegative matrix factorization using target-to-background contrast for fluorescence unmixing*, 2013 IEEE International Conference on Medical Imaging Physics and Engineering, 19-20 Oct. 2013; 2013; pp 287-292.

11. Hantao, L. W.; Aleme, H. G.; Pedroso, M. P.; Sabin, G. P.; Poppi, R. J.; Augusto, F., Multivariate curve resolution combined with gas chromatography to enhance analytical separation in complex samples: A review. *Analytica Chimica Acta* **2012,** *731*, 11-23.

12. Prats-Mateu, B.; Felhofer, M.; de Juan, A.; Gierlinger, N., Multivariate unmixing approaches on Raman images of plant cell walls: new insights or overinterpretation of results? *Plant Methods* **2018,** *14* (1), 52.

13. Gut, Y.; Boiret, M.; Bultel, L.; Renaud, T.; Chetouani, A.; Hafiane, A.; Ginot, Y.-M.; Jennane, R., Application of chemometric algorithms to MALDI mass spectrometry imaging of pharmaceutical tablets. *Journal of Pharmaceutical and Biomedical Analysis* **2015,** *105*, 91-100.

14. Ma, Y.; Ye, S.; Zhao, D.; Liu, X.; Cao, L.; Zhou, H.; Zuo, G.; Shi, C., Using different matrix factorization approaches to identify muscle synergy in stroke survivors. *Medical Engineering & Physics* **2023,** *117*, 103993.

15. Yamada, S.; Ito, K.; Kurotani, A.; Yamada, Y.; Chikayama, E.; Kikuchi, J., InterSpin: Integrated Supportive Webtools for Low- and High-Field NMR Analyses Toward Molecular Complexity. *ACS Omega* **2019,** *4* (2), 3361-3369.

16. Braidy, N.; Gosselin, R., Unmixing noisy co-registered spectrum images of multicomponent nanostructures. *Scientific Reports* **2019,** *9* (1), 18797.

17. Decesari, S.; Finessi, E.; Rinaldi, M.; Paglione, M.; Fuzzi, S.; Stephanou, E. G.; Tziaras, T.; Spyros, A.; Ceburnis, D.; O'Dowd, C.; Dall'Osto, M.; Harrison, R. M.; Allan, J.; Coe, H.; Facchini, M. C., Primary and secondary marine organic aerosols over the North Atlantic Ocean during the MAP experiment. *Journal of Geophysical Research: Atmospheres* **2011,** *116* (D22).

18. Thiel, M.; Sauwen, N.; Khamiakova, T.; Maes, T.; Govaerts, B., Comparison of chemometrics strategies for the spectroscopic monitoring of active pharmaceutical ingredients in chemical reactions. *Chemometrics and Intelligent Laboratory Systems* **2021,** *211*, 104273.

19. Gendrin, C.; Roggo, Y.; Collet, C., Pharmaceutical applications of vibrational chemical imaging and chemometrics: A review. *Journal of Pharmaceutical and Biomedical Analysis* **2008,** *48* (3), 533-553.

20. Omidikia, N.; Ghaffari, M.; Jansen, J.; Buydens, L.; Tauler, R., Bilinear model factor decomposition: A general mixture analysis tool. *Chemometrics and Intelligent Laboratory Systems* **2023,** *240*, 104901.

21. Xie, C.; Huang, Z.; E, Y.; Zhang, X.-C.; Kang, X.; Ma, Y.; Huang, P.; Zhang, G., THz Spectroscopic Decomposition and Analysis in Mixture Inspection Using Soft Modeling Methods. *Journal of Infrared, Millimeter, and Terahertz Waves* **2021,** *42* (1), 76-92.

22. Barton, B.; Thomson, J.; Lozano Diz, E.; Portela, R., Chemometrics for Raman Spectroscopy Harmonization. *Applied Spectroscopy* **2022,** *76* (9), 1021-1041.

23. Bordet, P.; Kergourlay, F.; Pinto, A.; Blanc, N.; Martinetto, P., Applying multivariate analysis to X-ray diffraction computed tomography: the study of medieval applied brocades. *Journal of Analytical Atomic Spectrometry* **2021,** *36* (8), 1724-1734.

24. Huang, S.; Zhao, Y.; Qin, B., Two-hierarchical nonnegative matrix factorization distinguishing the fluorescent targets from autofluorescence for fluorescence imaging. *BioMedical Engineering OnLine* **2015,** *14* (1), 116.

25. Liu, Z.; Huang, M.; Zhu, Q.; Qin, J.; Kim, M. S., A packaged food internal Raman signal separation method based on spatially offset Raman spectroscopy combined with FastICA. *Spectrochimica Acta Part A: Molecular and Biomolecular Spectroscopy* **2022,** *275*, 121154.

26. Pedregosa, F.; Varoquaux, G.; Gramfort, A.; Michel, V.; Thirion, B.; Grisel, O.; Blondel, M.; Prettenhofer, P.; Weiss, R.; Dubourg, V., Scikit-learn: Machine learning in Python. *the Journal of machine Learning research* **2011,** *12*, 2825-2830.

27. Ando, M.; Hamaguchi, H.-o., Molecular component distribution imaging of living cells by multivariate curve resolution analysis of space-resolved Raman spectra. *Journal of biomedical optics* **2013,** *19* (1), 011016-011016.

28. Wang, Y. X.; Zhang, Y. J., Nonnegative Matrix Factorization: A Comprehensive Review. *IEEE Transactions on Knowledge and Data Engineering* **2013,** *25* (6), 1336-1353.

29. Ahmadi, G.; Tauler, R.; Abdollahi, H., Multivariate calibration of first-order data with the correlation constrained MCR-ALS method. *Chemometrics and Intelligent Laboratory Systems* **2015,** *142*, 143-150.

30. Li, Y.; Wang, R.; Fang, Y.; Sun, M.; Luo, Z., Alternating Direction Method of Multipliers for Convolutive Non-Negative Matrix Factorization. *IEEE Transactions on Cybernetics* **2022**, 1-14.

31. Garrido, M.; Rius, F. X.; Larrechi, M. S., Multivariate curve resolution–alternating least squares (MCR-ALS) applied to spectroscopic data from monitoring chemical reactions processes. *Analytical and Bioanalytical Chemistry* **2008,** *390* (8), 2059-2066.

32. Motegi, H.; Tsuboi, Y.; Saga, A.; Kagami, T.; Inoue, M.; Toki, H.; Minowa, O.; Noda, T.; Kikuchi, J., Identification of Reliable Components in Multivariate Curve Resolution-Alternating Least Squares (MCR-ALS): a Data-Driven Approach across Metabolic Processes. *Scientific Reports* **2015,** *5* (1), 15710.

33. Bayat, M.; Marín-García, M.; Ghasemi, J. B.; Tauler, R., Application of the area correlation constraint in the MCR-ALS quantitative analysis of complex mixture samples. *Analytica Chimica Acta* **2020,** *1113*, 52-65.

34. Smith, J. P.; Holahan, E. C.; Smith, F. C.; Marrero, V.; Booksh, K. S., A novel multivariate curve resolution-alternating least squares (MCR-ALS) methodology for application in hyperspectral Raman imaging analysis. *Analyst* **2019,** *144* (18), 5425-5438.

35. Smith, J. P.; Smith, F. C.; Booksh, K. S., Multivariate Curve Resolution–Alternating Least Squares (MCR-ALS) with Raman Imaging Applied to Lunar Meteorites. *Applied Spectroscopy* **2018,** *72* (3), 404-419.

36. Smith, J. P.; Smith, F. C.; Booksh, K. S., Spatial and spectral resolution of carbonaceous material from hematite (α-Fe_2O_3) using multivariate curve resolution-alternating least squares (MCR-ALS) with Raman microspectroscopic mapping: implications for the search for life on Mars. *Analyst* **2017,** *142* (17), 3140-3156.

37. Smith, J. P.; Smith, F. C.; Ottaway, J.; Krull-Davatzes, A. E.; Simonson, B. M.; Glass, B. P.; Booksh, K. S., Raman Microspectroscopic Mapping with Multivariate Curve Resolution–Alternating Least Squares (MCR-ALS) Applied to the High-Pressure Polymorph of Titanium Dioxide, TiO_2-II. *Applied Spectroscopy* **2017,** *71* (8), 1816-1833.

38. Smith, J. P.; Smith, F. C.; Krull-Davatzes, A. E.; Simonson, B. M.; Glass, B. P.; Booksh, K. S., Raman microspectroscopic mapping with multivariate curve resolution-alternating least squares (MCR-ALS) of the high-pressure, α-PbO_2-structured polymorph of titanium dioxide, TiO_2-II. *Chemical Data Collections* **2017,** *9*, 35-43.

39. Felten, J.; Hall, H.; Jaumot, J.; Tauler, R.; De Juan, A.; Gorzsás, A., Vibrational spectroscopic image analysis of biological material using multivariate curve resolution–alternating least squares (MCR-ALS). *Nature Protocols* **2015,** *10* (2), 217-240.

40. Ruckebusch, C.; Blanchet, L., Multivariate curve resolution: A review of advanced and tailored applications and challenges. *Analytica Chimica Acta* **2013,** *765*, 28-36.
